# Exploring the oncogenic roles of LINC00857 in pan-cancer

**DOI:** 10.3389/fphar.2022.996686

**Published:** 2022-09-08

**Authors:** Xiaomin Ren, Jing Liu, Rui Wang, Xinling Liu, Xiaolin Ma, Zhong Lu, Zhenbo Hu, Mingzhu Zheng, Jingang Ma, Jiaqiu Li

**Affiliations:** ^1^ Department of Oncology, Affiliated Hospital of Weifang Medical University, School of Clinical Medicine, Weifang Medical University, Weifang, Shandong, China; ^2^ Department of Hematology, Laboratory for Stem Cell and Regenerative Medicine, Clinical Research Center, Affiliated Hospital of Weifang Medical University, Weifang, Shandong, China

**Keywords:** LINC00857, biomarker, bioinformatics, tumor immunity, pan-cancer

## Abstract

Although aberrant LINC00857 expression may play a key role in oncogenesis, no research has analyzed the pan-cancer oncogenic roles of LINC00857, particularly in tumor immunology. Here, we integrated data from several databases to analyze the characteristics of LINC00857 in pan-cancer. We found that LINC00857 was overexpressed and correlated with a poor prognosis in a variety of cancers. Furthermore, high-expression of LINC00857 was negatively associated with immune cell infiltration and immune checkpoint gene expression. Notably, LINC00857 expression was negatively related to microsatellite instability and tumor mutation burden in colorectal cancer, implying poor reaction to immunotherapy when LINC00857 was highly expressed. Targeting LINC00857 could dramatically impair the proliferative ability of colorectal cancer cells. After RNA-sequencing in HCT116 cells, gene set enrichment analysis showed that LINC00857 may accelerate cancer progression by inhibiting the ferroptosis pathway and promoting glycolipid metabolism in colorectal cancer. Screening by weighted gene co-expression network analysis determined PIWIL4 as a target of LINC00857, which also performed an immunosuppressive role in colorectal cancer. Based on the structure of PIWIL4, a number of small molecule drugs were screened out by virtual screening and sensitivity analysis. In summary, LINC00857 expression was closely correlated with an immunosuppressive microenvironment and may be a novel diagnostic and prognostic biomarker for diverse cancers. The LINC00857/PIWIL4 axis may be predictive biomarkers for immunotherapy and valuable molecular targets for malignant tumors.

## Introduction

Cancer, a highly malignant disease, poses severe threats to human health worldwide. Despite ongoing efforts by scientists to ameliorate cancer treatment, mortality from cancer remains high (Sung et al., 2021). Conventional cancer treatments such as radiotherapy and chemotherapy have shown various limitations that can be attributed to the severe related side effects (Barazzuol et al., 2020; Bukowski et al., 2020). Consequently, with the increasing resistance to existing drugs, finding new therapeutic targets is urgently required.

In recent years, non-coding RNAs (ncRNAs) have intrigued numerous researchers. Moreover, ncRNAs may be potential therapeutic targets (Zhang et al., 2020). As emerging regulatory molecules, ncRNAs are instrumental in tumorigenesis, invasion, metastasis and drug resistance to cancer. For instance, lncRNA FEZF1-AS1 was reported to facilitate the proliferation and metastasis in colorectal cancer (Bian et al., 2018). The lncRNA GBCDRlnc1 was also found to induce chemoresistance in advanced gallbladder cancer (Cai et al., 2019). Similarly, LINC00857 was discovered to play a carcinogenic role in lung, liver, pancreatic and bladder cancers (Dudek et al., 2018; Xia et al., 2018; Han et al., 2020; Meng et al., 2021). LINC00857 likely acts as an oncogene contributing to cancer development. Nevertheless, most studies on LINC00857 have been restricted to one specific cancer type. Pan-cancer analysis has brought us to a new phase of cancer research in recent years. The commonalities and differences derived from the pan-cancer analysis help us to investigate the potential carcinogenic mechanisms and forecast the treatment results. Thus, it is valuable to delve into the oncogenic roles of LINC00857 in pan-cancer, providing novel orientations and tactics for the diagnosis and treatment of cancers.

Recently, the importance of immunotherapy in different cancer types has been highlighted. Although immunotherapy has some deficiencies, it is considered as a promising approach to combat diverse cancers (Sharma and Allison, 2015b; Kennedy and Salama, 2020). Several studies have shown that the cancer phenotype is mediated through the intrinsic activity of cancer cells and the complicated interplay of diverse cell categories in the tumor microenvironment, in particular, tumor-infiltrating immune cells (Candido and Hagemann, 2013). The most promising way to stimulate the immune system is to block immune checkpoint genes, which have displayed vigorous anti-tumor effects in the treatment of various malignant tumors (Sharma and Allison, 2015a; Postow et al., 2015). Tumor mutation burden (TMB) has been validated as a biomarker of immune checkpoint inhibition response in multiple cancers (Yarchoan et al., 2017; Yarchoan et al., 2019). Tumor genomic mutations cause the development of neoantigens (NEO) that may be effective targets for anti-tumor immunotherapy (Blass and Ott, 2021). The high reaction rate to blocking immune checkpoint genes in patients with microsatellite instability (MSI) further confirmed the effect of neoantigens in the anti-tumor immunotherapy (Schrock et al., 2019). Conversely, cancers with poor NEO, MSI and TMB may be hyposensitive to immune checkpoint blockade. Nevertheless, current reports on the association between LINC00857 expression and immunological features are scarce.

In the present analysis, we elucidated the expression, prognostic value and immune characteristics of LINC00857 in pan-cancer using bioinformatics. Our study found that LINC00857 accelerated the proliferation of colorectal cancer cells, which may offer a potential therapeutic target for colorectal cancer. We explored the possible regulatory mechanisms by RNA-sequencing analysis.

## Materials and methods

### Assistant for clinical bioinformatics database analysis

ACLBI tool (https://www.aclbi.com/) is an integrated online platform for bioinformatics analysis. The LINC00857 gene expression levels and correlation analysis with immune cell infiltration or immune checkpoint genes expression were discussed using the “pan-cancer single gene fast comprehensive analysis” module. Correlation was measured by Spearman analysis.

### Lnc2Cancer 3.0 database analysis

Lnc2Cancer 3.0 (Gao et al., 2021) is an online database including comprehensive experimental supported lncRNAs or circRNAs in human cancers. In the “RNA-seq Web Tools” module, we selected the “Box Plot” and “Stage Plot” module to perform a search for the expression levels of LINC00857 in different cancers datasets using the Kruskal-Walli’s test. “Survival” module was implemented for disease free survival (DFS) or overall survival (OS) analysis based on LINC00857 expression and the median was chosen as the group cutoff for survival curve. *p* value cutoff is 0.05.

### StarBase V3.0 database analysis

The StarBase V3.0 (Li et al., 2014) online website was utilized to assess the expression of LINC00857 between cancers and matched normal samples. Additionally, the platform also predicted the correlation between PIWIL4 and CD274 (PD-L1, programmed cell death 1 ligand 1), PD1 (PDCD1, programmed cell death 1) and CTLA4 (cytotoxic T-lymphocyte associated protein 4) expression in COAD. *p* value cutoff is 0.05.

### GEPIA2 database analysis

The GEPIA2 data pool (Tang et al., 2019) was utilized to probe the expression and survival status in cancers and normal tissues. Survival curves of OS and DFS for LINC00857 expression were displayed by selecting the “Survival Plot” module under the condition of group cutoff = median. In the “Expression DIY” module, we adopted box plots to exhibit the expression status of LINC00857 in diverse cancer stages. The relationship between LINC00857 and PIWIL4 expression in colorectal cancer was explored in the “Correlation Analysis” pane. *p* value cutoff is 0.05.

### Kaplan-Meier Plotter database analysis

Pan-cancer survival analysis about recurrence-free survival (RFS) and OS for LINC00857 expression was executed using the “mRNA-RNA-seq” module in the Kaplan Meier plotter website (Nagy et al., 2021). *p* value cutoff is 0.05.

### Sangerbox 3.0 database analysis

The Sangerbox 3.0 (http://vip.sangerbox.com/home.html) is a complimentary online platform for comprehensive bioinformatics analysis. The “Pan-cancer analysis tool” in the platform was implemented to explore the expression and immune characteristics of LINC00857 in various cancers. Concretely, the data source was set to “TCGA + GTEx” and the data transformation was set to “log_2_ (x+1)”. The correlation between all parameters were performed using the Pearson method. Subsequently, weighted gene co-expression network analysis (WGCNA) was implemented using raw counts through the “WGCNA analysis tool”. The soft threshold power of *β* = 7 (R2 = 0.86) was selected to assure a scale-free network. Next, the minimum number of modules was fixed at 30, the sensitivity at 2, the module merge threshold at 0.5 and other parameters were the default values for the site. Totally, 13 non-gray modules were derived. Additionally, functional enrichment analysis of 76 LINC00857-associated coding genes was implemented by the “gene ontology (GO) and kyoto encyclopedia of genes and genomes (KEGG) analysis tool”. The top 10 markedly enriched pathways (*p* < 0.05) were displayed according to gene count.

### CAMOIP database analysis

CAMOIP is an integrated web server for the analysis of pan-cancer immunotherapy (Lin et al., 2022). The “Immunogenicity”, “Immune Infiltration” as well as “Pathway Enrichment” modules in the database were used to explore the immune characteristics and gene set enrichment analysis (GSEA) of PIWIL4 in colon cancer.

### Gene set cancer analysis database analysis

GSCA is a comprehensive online tool for immunogenomic and pharmacogenomic cancer analysis (Liu et al., 2018). The “immune cell abundance” module was used to demonstrate the interrelation between PIWIL4 expression and immune cell infiltration in colon cancer by GSVA score. In addition, the relevance between PIWIL4 expression and GDSC/CTRP drug sensitivity was analyzed.

### Cell miner database analysis

Through the Cell Miner database (Reinhold et al., 2012), we obtained the gene expression profile of NCI-60 human cancer cell lines and the drug data approved by FDA or clinically verified. Next, the relevance analysis between PIWIL4 expression and drug sensitivity (IC50) was conducted by R software. The correlation score was calculated by Pearson coefficient and *p* value < 0.05.

### Molecular docking

Firstly, the 3D structure of PIWIL4 protein was obtained from Alpha Fold online platform (Jumper et al., 2021). Meanwhile, the PubChem database was used to download the chemical structure information of the drugs (Kim et al., 2021). Next, the possible small molecule binding sites on PIWIL4 protein surface were detected by GHECOM algorithm (Kawabata, 2010). The maximum pocket to cover the ligand was chosen, with a volume of 41,065 Å3. Lastly, molecular docking was performed using DOCK 6.9 software and the spatial conformation of the docking was visualized by PyMol software. The interaction between protein and small molecule drugs was calculated by ligplus. Grid_Score represented the total molecular docking score between the drug and protein. A smaller score value indicated stronger binding ability. Internal energy repulsion was usually less than 20.

### Cell culture

The human colorectal cancer cell-HCT116 was purchased from the cell bank of the Chinese Academy of Sciences. HCT116 cell were incubated in McCoy’s 5A medium (GNM16600, GENOM) complemented with 10% bovine serum at 37°C with 5% CO2.

### SiRNA transfection

HCT116 cells was conducted in 6-well plates (1 × 10^5^ cells) and transfected with siRNA transfection reagent (13778150, Invitrogen). The siRNA for transient knockdown of LINC00857 was obtained from Gene Pharma (Shanghai, China). The siRNA sequence showed below:

Negative control: S: UUC​UCC​GAA​CGU​GUC​ACG​UTT, AS: ACG​UGA​CAC​GUU​CGG​AGA​ATT; LINC00857-siRNA1#: S: GGC​UAU​GUG​CUG​UGA​ACA​ATT, AS: UUG​UUC​ACA​GCA​CAU​AGC​CTT; LINC00857-siRNA2#: S: GGU​AUU​AGU​GGG​UGA​AUA​UTT, AS: AUA​UUC​ACC​CAC​UAA​UAC​CTT.

### RNA isolation and quantification

All RNA isolation was performed utilizing the Trizol (Qiagen, 1023537). Inverse transcription was accomplished with the cDNA Synthesis Kit (4375222, AB). The real-time qPCR was implemented by SYBR Green fluorescence measurement. β-actin was used as the normalized endogenous control. The primer sequences utilized were listed below:

PIWIL4-F: AAG​CCC​ACA​CAC​ACC​TTT​CA, R: TGG​TCA​GTC​AGC​CCT​GTT​AG; LINC00857-F: AGA​ACG​CGG​TGT​GAA​GGA​AA, R: TGA​GCC​CTG​GGA​AAC​AAT​GA; actin-F: CAC​CAA​CTG​GGA​CGA​CAT, R: ACA​GCC​TGG​ATA​GCA​ACG.

### RNA sequencing analysis

RNA sequencing was performed by Lianchuan Biotechnology Co. (Hangzhou, China). GSEA analysis was conducted by GSEA (v4.1.0) and MSigDB (Subramanian et al., 2005). In short, the genes were sorted using the Signal2Noise normalization method. GO terms and KEGG pathways fulfilling the conditions with |NES|>1 and NOM p-val<0.05 were deemed to be significant in the two groups. Subsequently, downstream analysis was implemented using raw counts of the genes in the selected modules. The R package “limma” was employed to perform normalized and differential expression analysis (Ritchie et al., 2015). Differentially expressed genes (DEGs) were chosen with the following screening criteria: *p* < 0.05, log_2_|fould change|>1. Combining the two sets of different analysis data, 76 candidate genes were finally obtained after excluding non-coding genes. The volcano plots and heatmap were created by the online data visualization platform-bioinformatics (http://www.bioinformatics.com.cn). The LINC00857-miRNA-PIWIL4 interaction network diagram was constructed using Cytoscape (v3.9.0).

### Colony formation assays

Colony formation assays were determined to assess colony formation ability. 1,000 cells per well were sowed and incubated in six-well plates in triplicate. 11 days after cultivation, colonies were dyed with 0.1% crystal violet and then counted.

### CCK8 assay

The effect of LINC00857 on cell viability was detected by CCK8 kit (C0038-500, Beyotime). After transfection, 3,000 cells each well were transferred into 96-well plates for 72 h, then CCK8 working solution was pipetted into every well and cultured in medium at 37°C for 30 min. Finally, absorbance (optical density, OD) was gauged at 450 nm by a microplate reader.

### Fluorescence *in Situ* hybridization

FISH assays were proceeded as standard programs. The sample slides for the FISH experiments were fixed with 4% formaldehyde. Briefly, tissue or cell slides were dewaxed, dehydrated, hybridized, washed and counterstained with green fluorescence for LINC00857 expression (FAM (488)). LINC00857 FISH probe was manufactured by Servicebio (Wuhan, China) and its sequence was presented as below: 5′-FAM-TTGGGACAGGGTTTGGAACTCTTGCGG-FAM-3’.

### Statistics

Student’s t-test, one-way or two-way ANOVA were administered to assess statistical significance. *p* < 0.05 were indicated statistically significant.

## Results

### Expression levels of LINC00857 in pan-cancer

**FIGURE 1:**
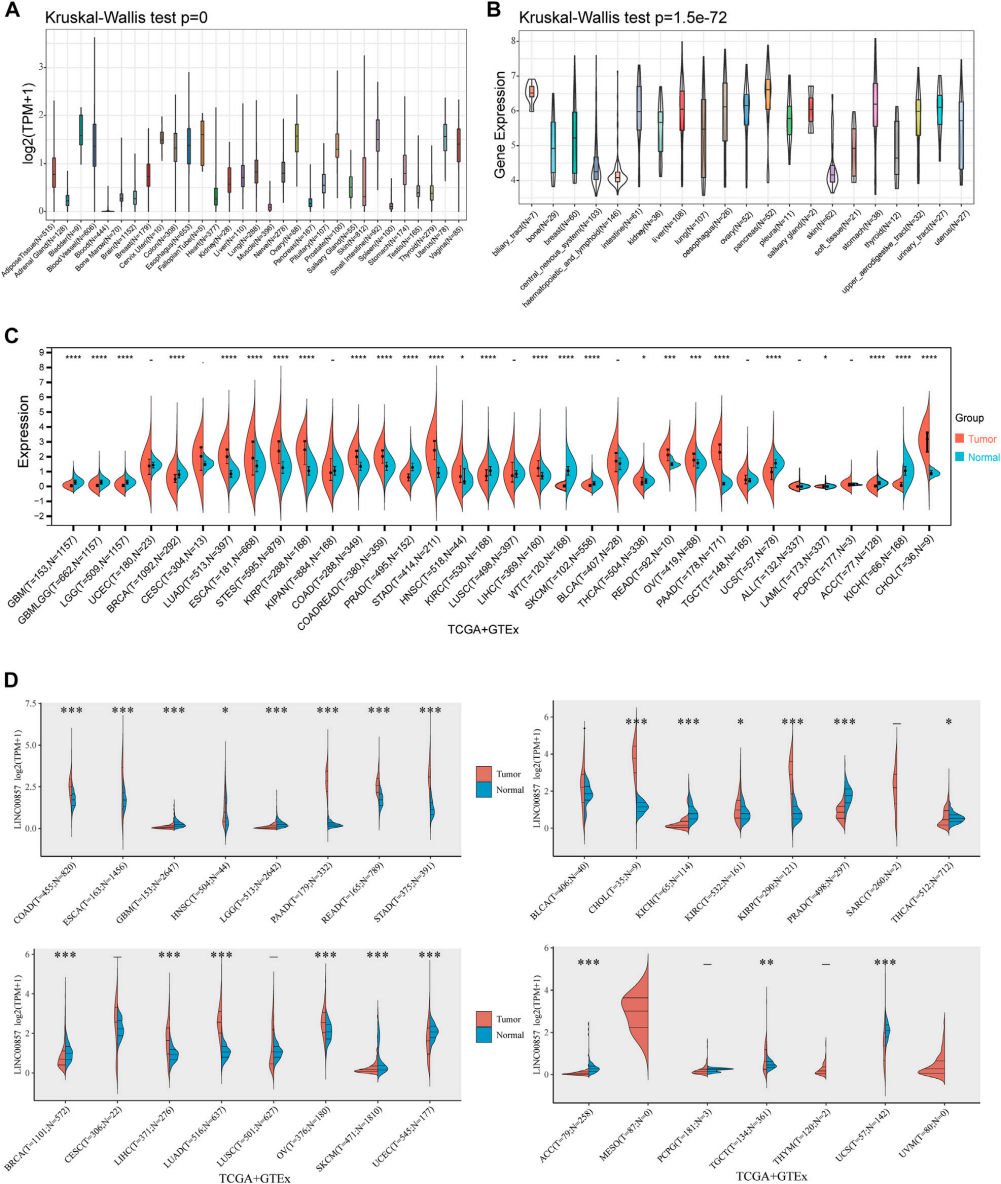
The expression levels of LINC00857 in pan-cancer. **(A)** LINC00857 expression in 31 human normal tissues from GTEx database. **(B)** LINC00857 expression in various cancer cell lines from CCLE database. **(C)** The expression levels of LINC00857 in different cancers and adjacent normal tissues were explored through Sangerbox 3.0 database. **(D)** The expression levels of LINC00857 in cancers or paired normal tissues were analyzed *via* online website ACLBI (*p < 0.05, **p < 0.01, ***p < 0.001).

To ascertain the expression levels of LINC00857 in normal and cancers samples, we obtained data from TCGA, CCLE and GTEx datasets. We explored the expression levels of LINC00857 in 31 healthy tissues and 21 cancer cell lines through the Sangerbox database. In normal samples, the highest expression level of LINC00857 was observed in bladder tissue and the lowest in blood (Kruskal–Wallis’s test *p* < 0.001) (Figure 1A). By contrast, LINC00857 was highly expressed in most cancer cell lines, which may imply its effect on the malignant phenotype of cancer cells (Figure 1B). As shown in Figures 1C,D and Supplementary Figure S1, we characterized the expression levels of LINC00857 in cancers and corresponding normal samples using the TCGA and GTEx datasets. By simultaneous analysis of four databases (Sangerbox 3.0, ACLBI, StarBase V3.0 and Lnc2Cancer 3.0), we found that the expression levels of LINC00857 were markedly higher in eight cancers than in the adjacent normal tissues including HNSC, CHOL, COAD, KIRP, STAD, LIHC, PAAD and LUAD. LINC00857 expression was obviously decreased in BRCA, KICH, KIRC and PRAD. Furthermore, stage plot in the Lnc2Cancer 3.0 and GEPIA2 databases suggest that LINC00857 expression was strikingly correlated with the cancer pathological stages (Supplementary Figure S2). Consequently, these results support that LINC00857 may act as a new diagnostic biomarker for many cancer types.

### The prognostic value of LINC00857 in pan-cancer

**FIGURE 2:**
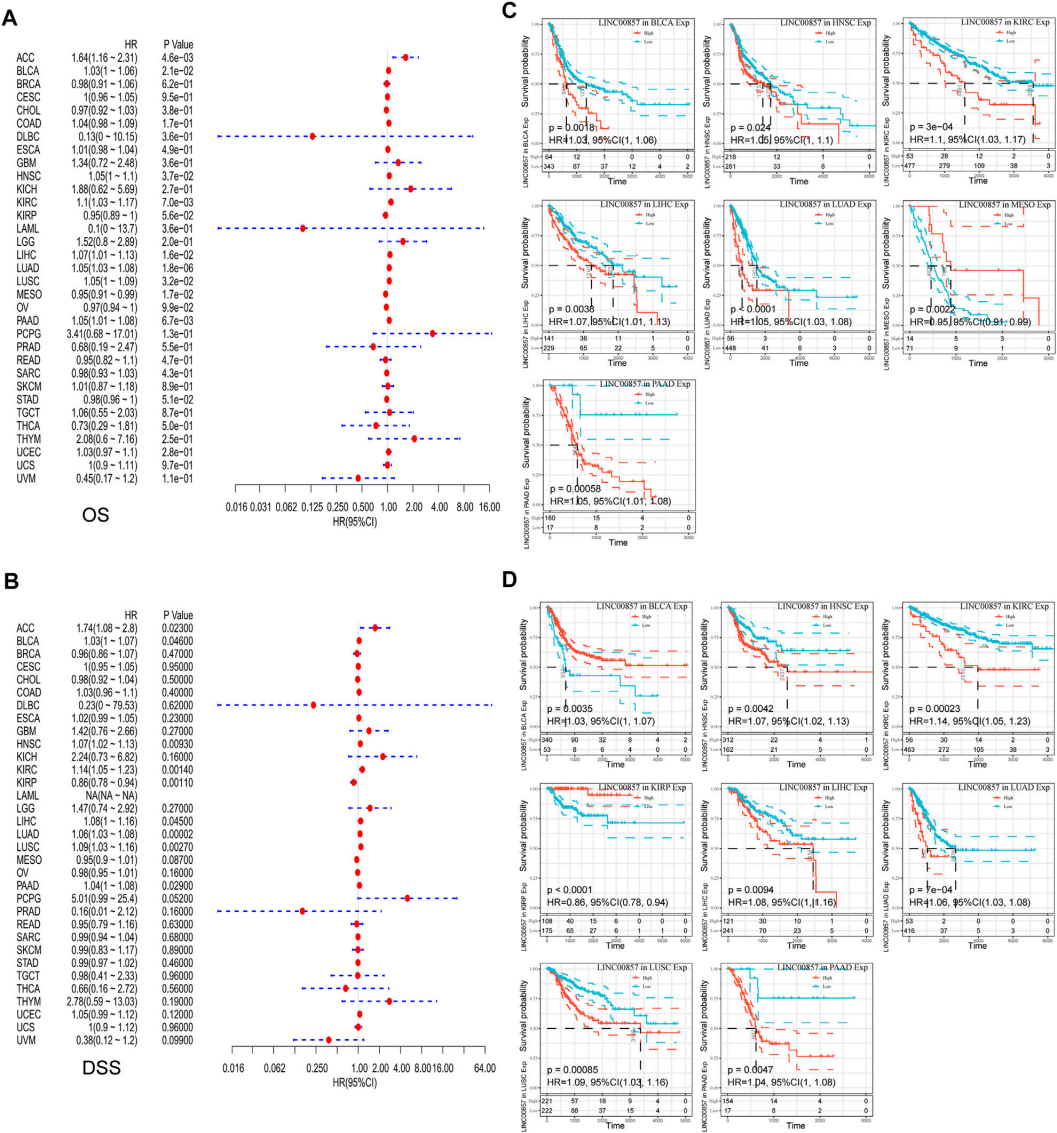
The correlation between LINC00857 expression and OS and DSS in pan-cancer. **(A,B)** Cox regression analysis of LINC00857 on OS and DSS for multiple neoplasms was depicted *via* forest diagrams. **(C,D)** Kaplan-Meier curves presented OS or DSS for patients with high and low LINCOO857 expression (*p* < 0.05).

**FIGURE 3:**
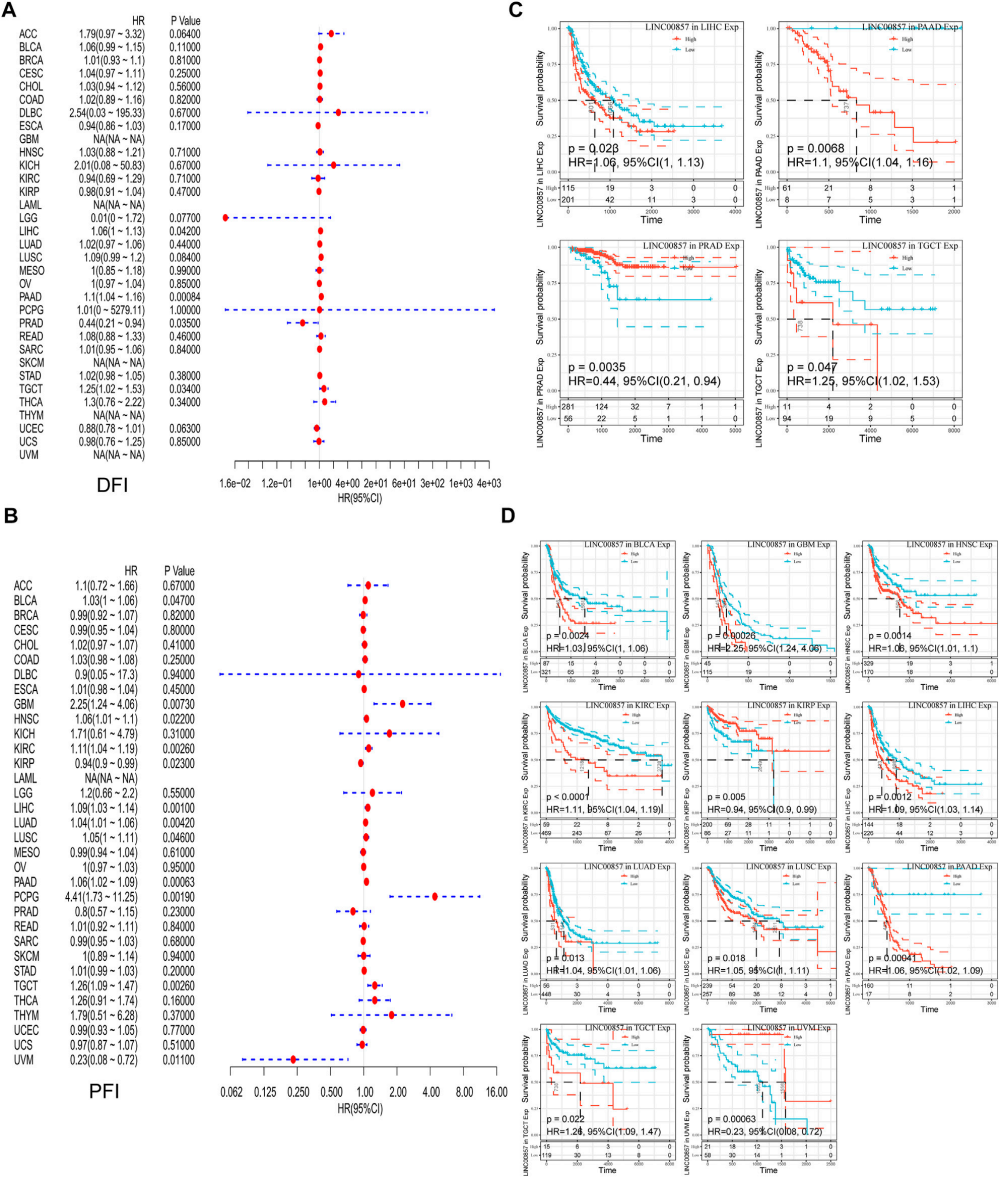
The correlation between LINC00857 expression and DFI and PFI in pan-cancer. **(A,B)** Cox regression analysis of LINC00857 on DFI and PFI for different neoplasms was depicted by forest diagrams. **(C,D)** Kaplan-Meier curves presented the prognostic value of LINC00857 for DFI or PFI (*p* < 0.05).

To illustrate the relevance between LINC00857 gene expression and prognosis, survival analysis for LINC00857 among different cancers was performed using Sangerbox, GEPIA2, Kaplan-Meier Plotter and Lnc2Cancer 3.0 databases. First, Cox regression analysis was implemented for 33 cancer types by TCGA datasets. The association between LINC00857 expression and OS, disease-specific survival (DSS), disease-free interval (DFI) and progression-free interval (PFI) was displayed in forest plots (Figures 2A,2B, 3A,3B). Based on these results, we further elaborated the prognostic value of LINC00857 in these cancers using Kaplan-Meier (KM) analysis (Figures 2C,D, 3C,D). We observed that higher LINC00857 expression was related to poorer OS/DSS/PFI in BLCA, HNSC, KIRC, LIHC, LUAD, PAAD, and with poorer DFI in TGCT, PAAD, LIHC. Nevertheless, patients with high LINC00857 displayed markedly longer OS in MESO, longer DSS in KIRP, longer DFI in PRAD, as well as longer PFI in KIRP and UVM. In GEPIA2 and Kaplan-Meier Plotter databases, LINC00857 hyperexpression was positively correlated with poor OS/DFS/RFS in LIHC and PAAD, but only with poor OS in LUAD (Supplementary Figures S3A,B). In the Lnc2Cancer 3.0 database, higher LINC00857 expression was related to poor OS in LUAD, poor OS/DFS in PAAD and poor DFS in LIHC and GBM (Supplementary Figure S3C). In conclusion, these data validated that LINC00857 expression was highly associated with the prognosis in multiple cancers, implicating that LINC00857 may be a reasonable biomarker to evaluate the prognosis of many cancers.

### The relevance between LINC00857 expression and immune cell infiltration

**FIGURE 4:**
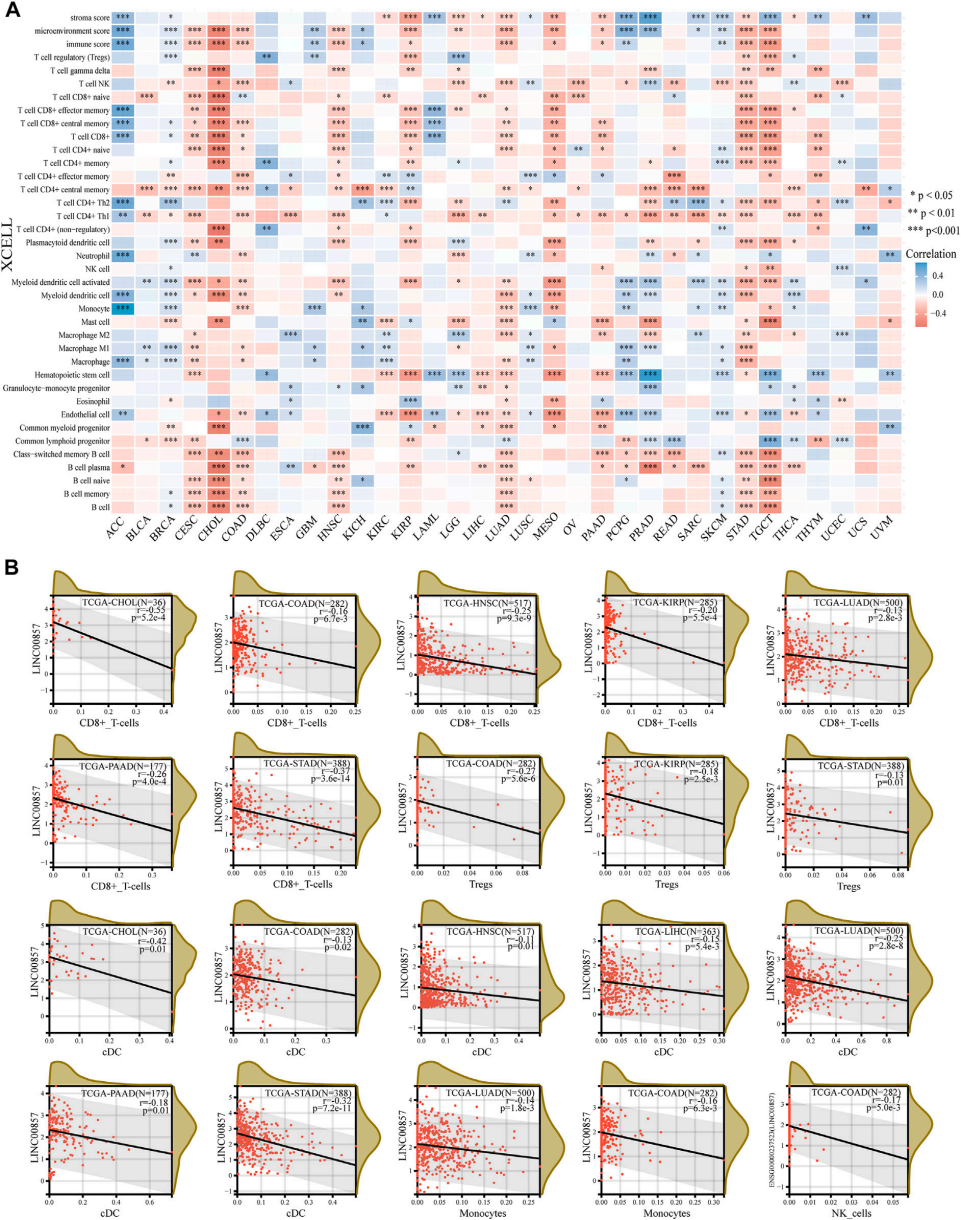
The correlation between LINC00857 expression and immune cell infiltration. **(A)** The interrelation heatmap between LINC00857 expression and immune cell infiltration levels was examined by the ACLBI database (XCELL algorithm). (*p < 0.05, **p < 0.01, ***p < 0.001, Spearman analysis) **(B)** Scatter plots of the pertinence between LINC00857 expression and the infiltration levels of monocyte, NK cell, conventional DC, Treg and CD8+T cell by the Sangerbox 3.0 database (XCELL algorithm) (*p < 0.05, **p < 0.01, ***p < 0.001, Pearson analysis).

Tumor-infiltrating immune cells (TIICs), a significant portion of the tumor microenvironment, are intimately linked to tumorigenesis and cancer progression (Domingues et al., 2016). Thereby, we investigated the possible relevance between LINC00857 expression and TIICs in pan-cancer by Sangerbox 3.0 and ACLBI database. The heatmap detailing the interrelation between LINC00857 expression and TIICs was shown in Figure 4A. We further analyzed the relevance between LINC00857 expression and CD8^+^ T cells, NK cells, conventional dendritic cells (cDCs), Tregs and monocytes in eight LINC00857 high-expression cancers (Figure 4B). LINC00857 expression had a statistically inverse association with the immune cells in these eight cancers. Intriguingly, LINC00857 expression was inversely correlated with the infiltration levels of all five immune cells in COAD. In addition, the present research noticed that LINC00857 expression was negatively related to the immune score and microenvironment score in these eight cancers (Supplementary Figure S4). Overall, these findings indicated that LINC00857 expression was negatively associated with immune cell infiltration.

### The relevance between LINC00857 and immune checkpoint gene expression

**FIGURE 5:**
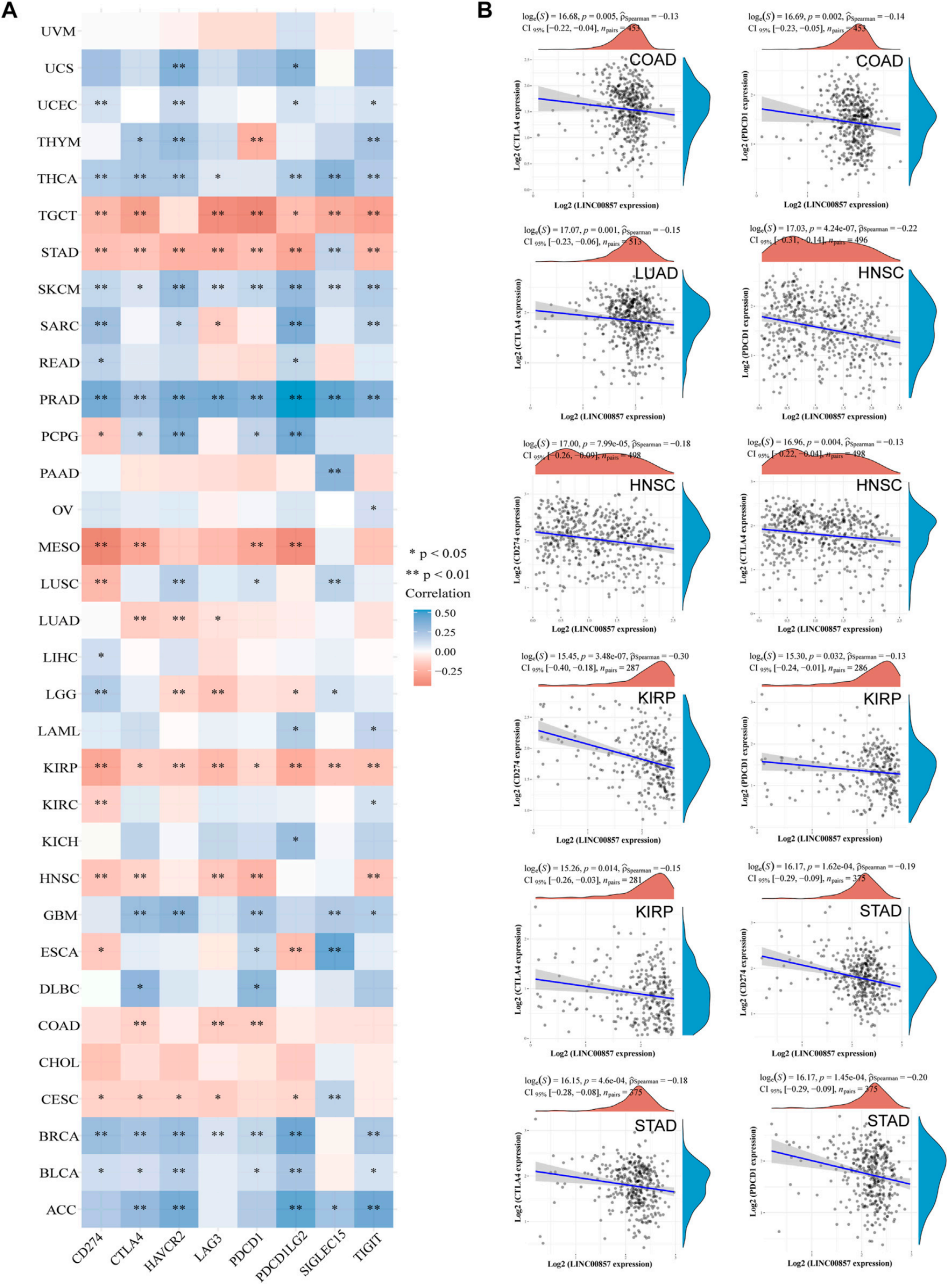
The correlation between LINC00857 and immune checkpoint genes expression. **(A)** Correlation mapping of LINC00857 expression with immune checkpoint genes in pan-cancer by the ACLBI database. **(B)** The correlation between LINC00857 and PD-L1(CD274), PD1 (PDCD1) or CTLA4 expression was shown *via* the scatter plot (*p < 0.05, **p < 0.01, ***p < 0.001, Spearman analysis).

Cancers can elude immune response by exploiting immune checkpoint genes such as PD-1/PD-L1 and CTLA-4. A promising immunotherapeutic strategy has emerged by targeting PD-1, PD-L1 or CTLA-4 to treat various malignancies (Postow et al., 2015). We studied the major immune checkpoint genes using the ACLBI database and analyzed their relevance to LINC00857 expression (Figure 5A). We observed a positive relevance between LINC00857 and most immune checkpoint gene expression in ACC, BLCA, BRCA, GBM, PRAD, SKCM and THCA, but negative relevance in TGCT, STAD, MESO, KIRP, COAD, HNSC, and CESC. Based on the above data, we further compared the relevance of PD-1, PD-L1 and CTLA-4 with LINC00857 expression in the above eight cancers. As depicted in Figure 5B, LINC00857 expression had a significant reverse correlation with PD-1, PD-L1 and CTLA-4 expression in these cancers. In brief, LINC00857 expression was closely associated with immune checkpoint gene expression.

### The relevance between LINC00857 expression and MSI, TMB or NEO

**FIGURE 6:**
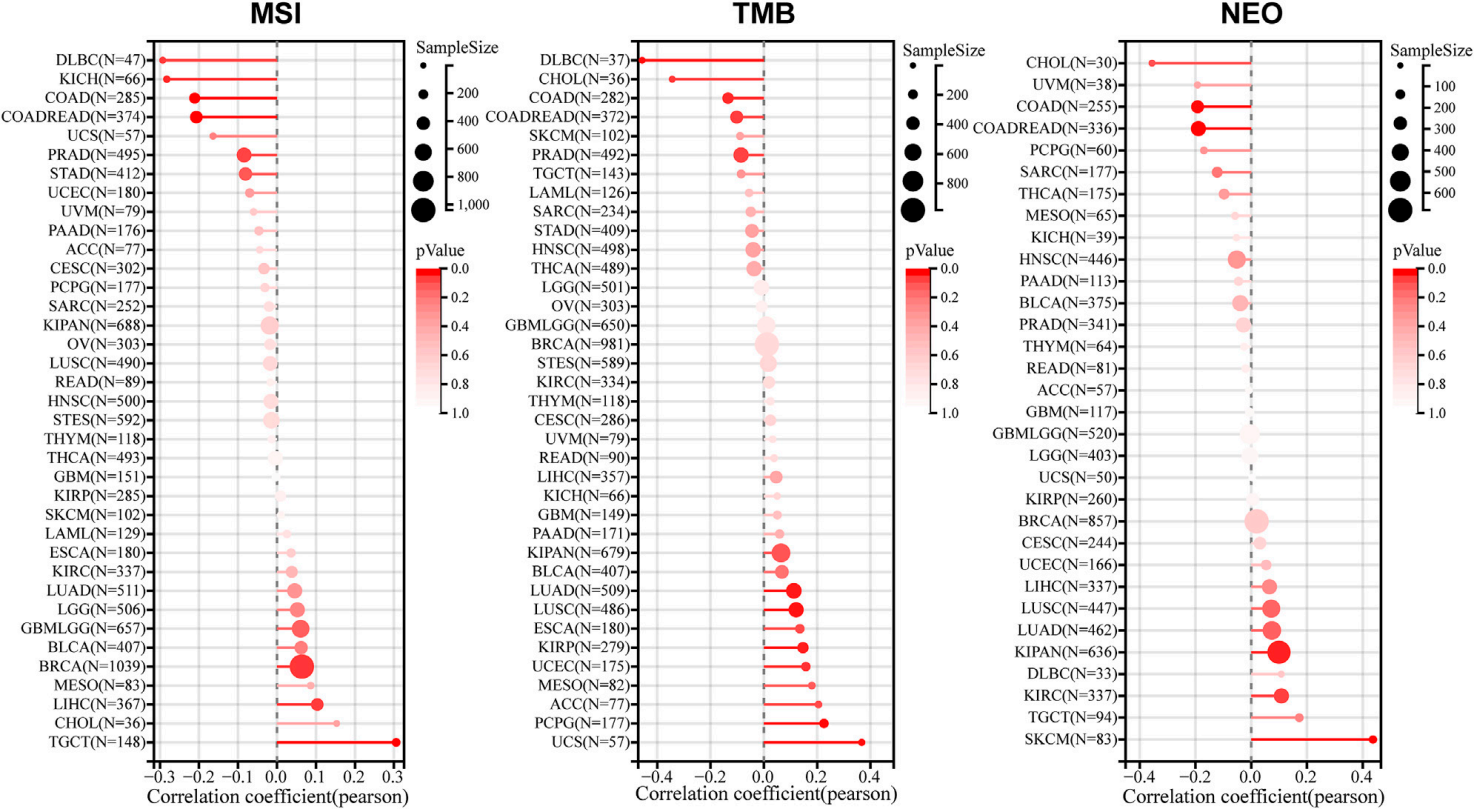
The correlation between LINC00857 expression and MSI, TMB or NEO. A stick chart displayed the connections between LINC00857 expression and MSI, TMB or NEO in diverse cancers *via* the Sangerbox 3.0 database (Pearson correlation).

In the tumor microenvironment, MSI, TMB and NEO are correlated with anti-tumor immunity and serve as valid biomarkers of tumor immunotherapeutic response (Dudley et al., 2016; Yarchoan et al., 2017; Peng et al., 2019). To validate the significance of LINC00857 expression in immunotherapy, a pan-cancer assessment was performed for MSI, TMB and NEO (Figure 6). We discovered that LINC00857 expression was dramatically associated with MSI in seven cancers, with a positive correlation in three cancers including BRCA, LIHC, TGCT and a negative correlation in COAD, COADREAD, KICH, DLBC. LINC00857 expression was dramatically associated with TMB in ten cancers, with a positive correlation in six cancers including LUAD, KIRP, UCEC, LUSC, PCPG, UCS and a negative correlation in COAD, COADREAD, CHOL, DLBC. LINC00857 expression was positively associated with NEO in KIPAN and SKCM while was negatively correlated with in COAD and COADREAD. Intriguingly, among the eight malignancies mentioned above, LINC00857 expression was only simultaneously negative with TMB, MSI or NEO in colorectal cancer. In accordance with the above conclusion, it might hint a poor response to immunotherapy in colorectal cancer patients with high-expression of LINC00857.

### LINC00857 promoted the proliferation of colorectal cancer cells

**FIGURE 7:**
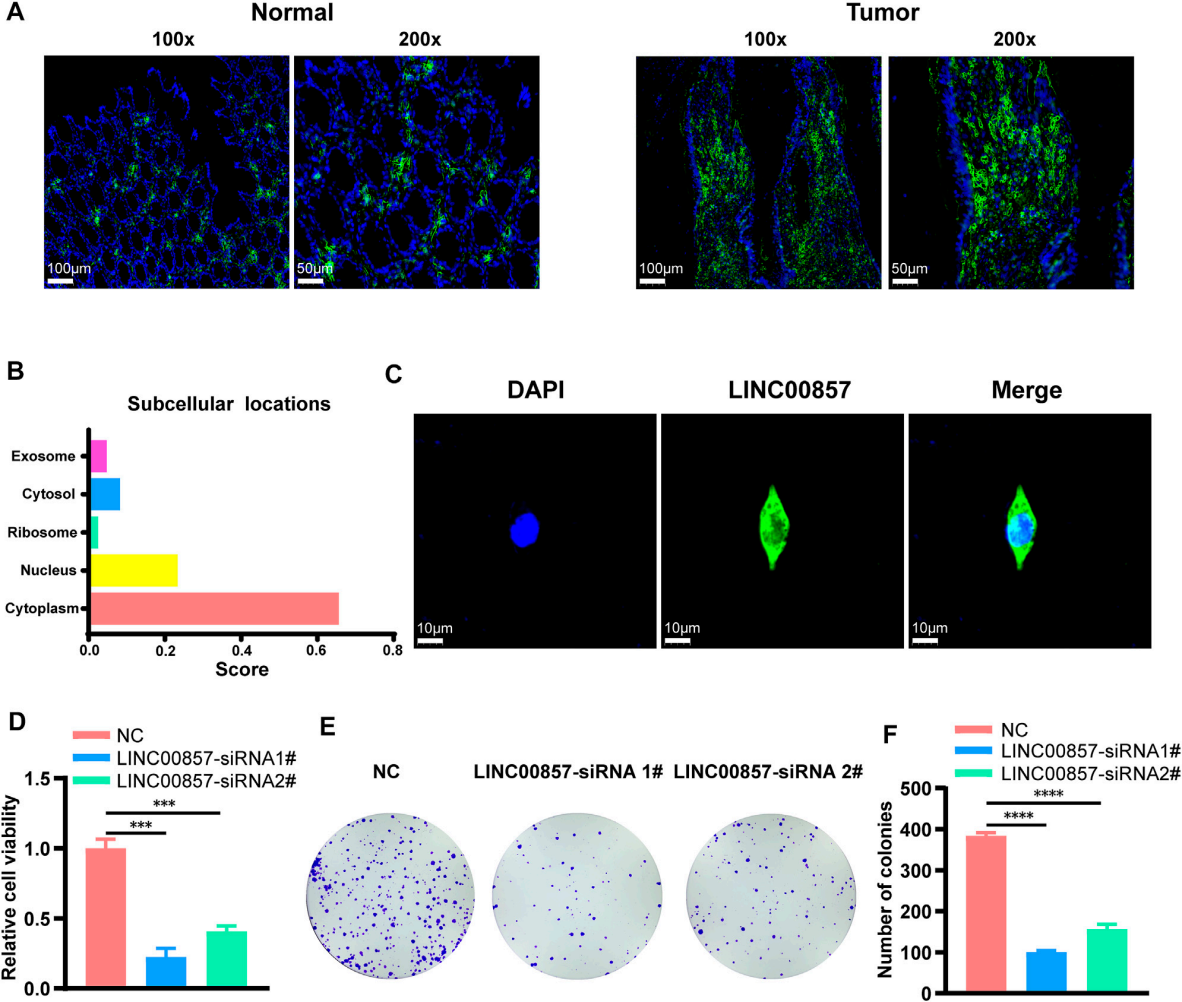
LINC00857 promoted the proliferation of colorectal cancer cells. **(A)** LINC00857 expression in colorectal cancer and corresponding normal tissues was examined by FISH assay. **(B)** The subcellular localization of LINC00857 was predicted by lncLocator tool. **(C)** The subcellular localization of LINC00857 in HCT116 cell was examined by FISH assay. **(D)** The cell viability of HCT116 cells transfected with siRNA-LINC00857 or siRNA-NC was evaluated by CCK-8 assay. **(E–F)** Colony formation experiments monitored the proliferative ability of HCT116 cells after LINC00857 silencing (*p < 0.05; **p < 0.01; ***p < 0.001).

Considering that colorectal cancer patients with high-expression of LINC00857 may not be suitable for immune checkpoint inhibitors, targeting LINC00857 may be an ideal approach for these patients. The FISH assays confirmed that LINC00857 expression was more widely expressed in colorectal cancer than in the normal tissues (Figure 7A). In general, the action of lncRNA is prominently interrelated to its subcellular localization (Chen, 2016). We found that LINC00857 was primarily distributed in the cytoplasm as elucidated by lncLocator tool (Cao et al., 2018) and FISH assays (Figures 7B,C). Subsequently, we designed two specific siRNAs against LINC00857 and transfected them into HCT116 colorectal cancer cells. To estimate the potential influence of LINC00857 silencing on colorectal cancer cell proliferation, we performed CCK-8 and colony formation experiments. Our results validated that knockdown of LINC00857 attenuated the proliferative ability of HCT116 cells (Figures 7D–F). Thus, targeting LINC00857 may be feasible to impair the proliferation of colorectal cancer cells.

### GSEA after LINC00857 knockdown

**FIGURE 8:**
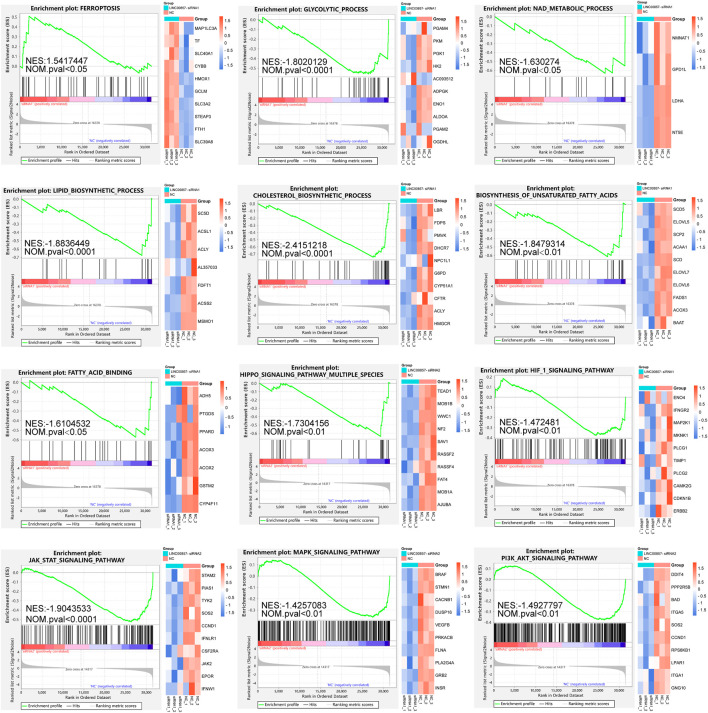
GSEA after LINC00857 silencing. Gene set enrichment analysis (GSEA) of the molecular signatures by RNA-sequencing in HCT116 cell after silencing LINC00857 (|NES|>1 and NOM *p* < 0.05).

For more insight into the regulatory mechanisms of LINC00857 in colorectal cancer, we conducted a transcriptome sequencing analysis after LINC00857 knockdown with two independent siRNAs in HCT116 cells. Afterwards, we executed GSEA to investigate the possible biological function of LINC00857 in colorectal cancer. As shown in Figure 8, GSEA findings revealed that knockdown of LINC00857 triggered the ferroptosis pathway, which might account for how LINC00857 facilitated cancer growth. Interestingly, downregulated genes after LINC00857 knockdown were remarkably enriched in multiple metabolic pathways such as glycolytic process, NAD metabolic process, lipid/cholesterol/unsaturated fatty acids biosynthetic process and fatty acid binding etc. Meanwhile, silencing LINC00857 affected the activation of numerous signaling pathways including Hippo, HIF1, JAK/STAT, MAPK and PI3K/AKT signaling pathways. These findings imply that LINC00857 may affect the proliferation of cancer cells *via* multiple biological pathways.

### Transcriptomic landscape after LINC00857 knockdown

**FIGURE 9:**
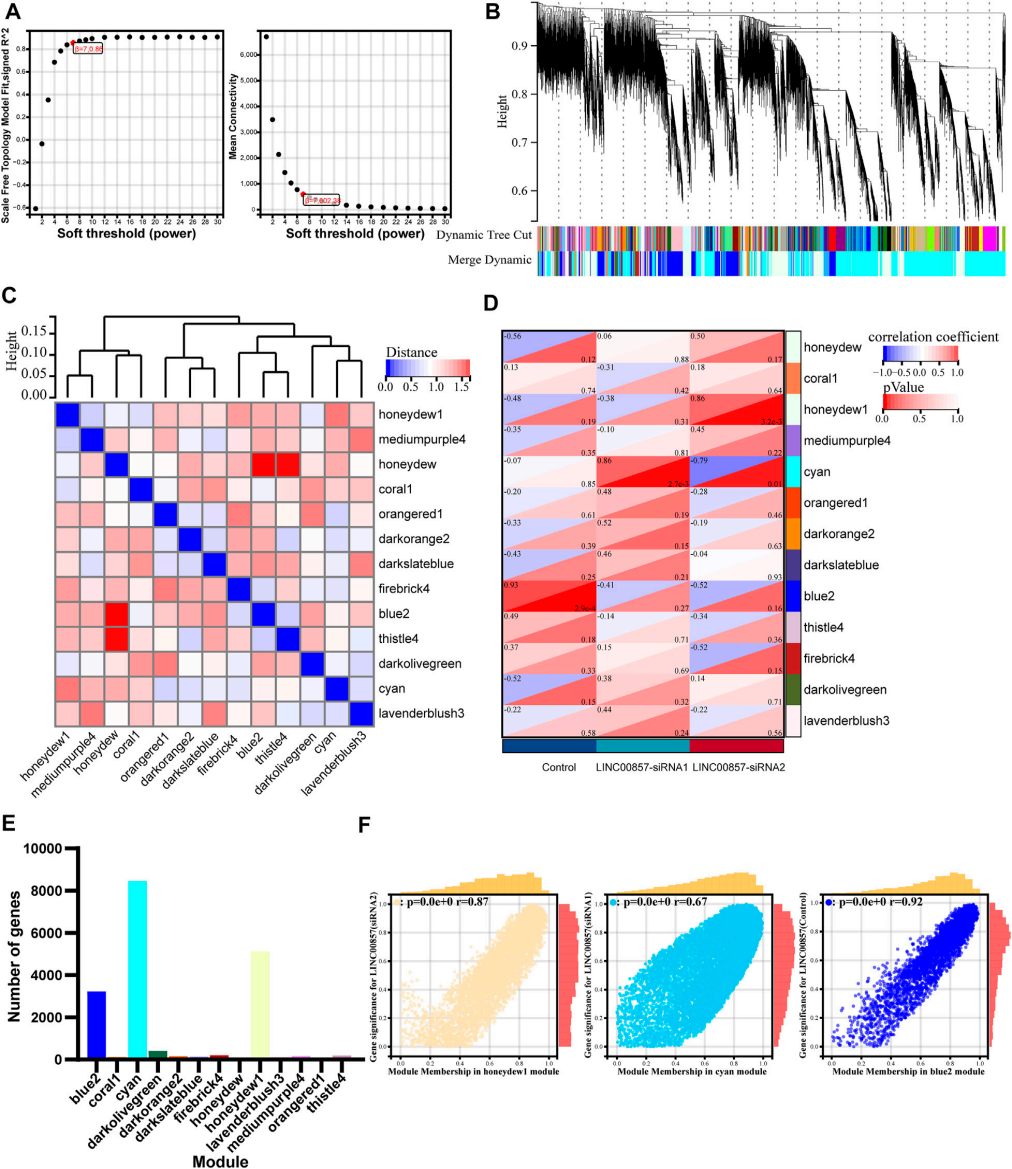
WGCNA after LINC00857 silencing. **(A)** The soft threshold of the WGCNA module. **(B)** Cluster dendrogram of LINC00857 RNA-seq expression data and functional modules were created by WGCNA. **(C)** The interaction between different modules. **(D)** Heatmap of interrelation between gene modules and sample characteristics. **(E)** The number of genes per module. **(F)** Dependence between GS and MM in honeydew 1, cyan and blue 2 modules. P< 0.05.

**FIGURE 10:**
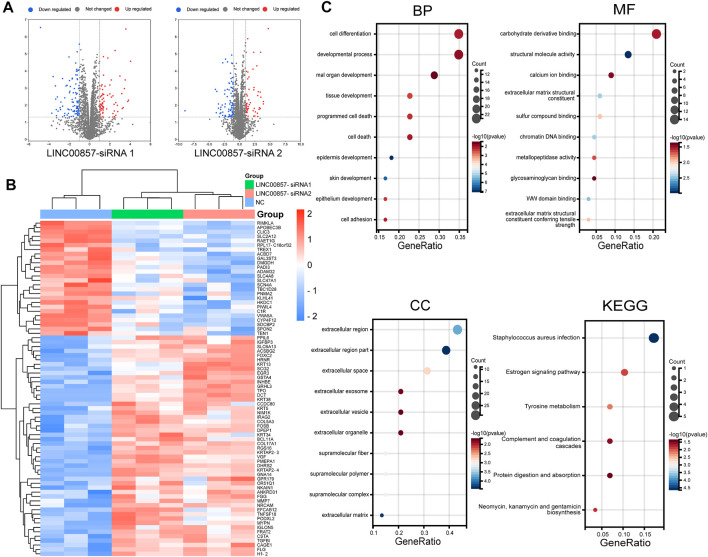
Transcriptomic landscape after LINC00857 silencing. **(A)** Volcano map depicted the distribution characteristics of all differential genes. (*p* < 0.05, |log_2_FC|>1) **(B)** Heatmap displayed the expression levels of 76 differentially expressed genes (DEGs). **(C)** GO and KEGG analysis for the 76 DEGs.

Next, we implemented WGCNA of the abundant RNA-seq data to determine molecules underlying the regulatory mechanism of LINC00857 in colorectal cancer. To ensure connectivity between genes fulfilled the scale-free network layout, we set the soft threshold power to 7 (R2 = 0.86) (Figure 9A). As illustrated in the hierarchical clustering tree in Figure 9B, genes with analogous biological functions were categorized into the same module with the module’s smallest size cutoff of 30 genes. After combining highly similar modules, we ultimately gained 13 co-expression modules with sizes ranging from 49 to 8,449 genes. The interactions between these modules were indicated in Figure 9C. Among the indicated modules, the honeydew 1, cyan and blue2 co-expression modules were most dramatically associated with the control or LINC00857 knockdown group (Figure 9D). Intriguingly, these three groups were also the modules with the highest proportion of gene counts (Figure 9E). In addition, scatter plots of interrelation between gene significance and module membership in the honeydew 1, cyan and blue 2 modules were also presented separately (Figure 9F). Subsequently, differential expression analysis was executed on these gene datasets of the three modules. The volcanic plots preliminarily displayed the overall distribution of all differential genes (Figure 10A). As illustrated in Figure 10B, we identified 50 obviously upregulated mRNAs and 26 downregulated mRNAs. For a broader overview of LINC00857s role, we performed GO and KEGG analysis on these 76 candidate genes (Figure 10C). Cell differentiation, carbohydrate derivative binding, extracellular region and *Staphylococcus aureus* infection were remarkably enriched in biological process (BP), molecular function (MF), cellular component (CC) and KEGG pathways, respectively. These enriched pathways allowed us to better interpret the functions of LINC00857 in colorectal cancer.

### LINC00857 regulated the expression of PIWIL4 in colorectal cancer

**FIGURE 11:**
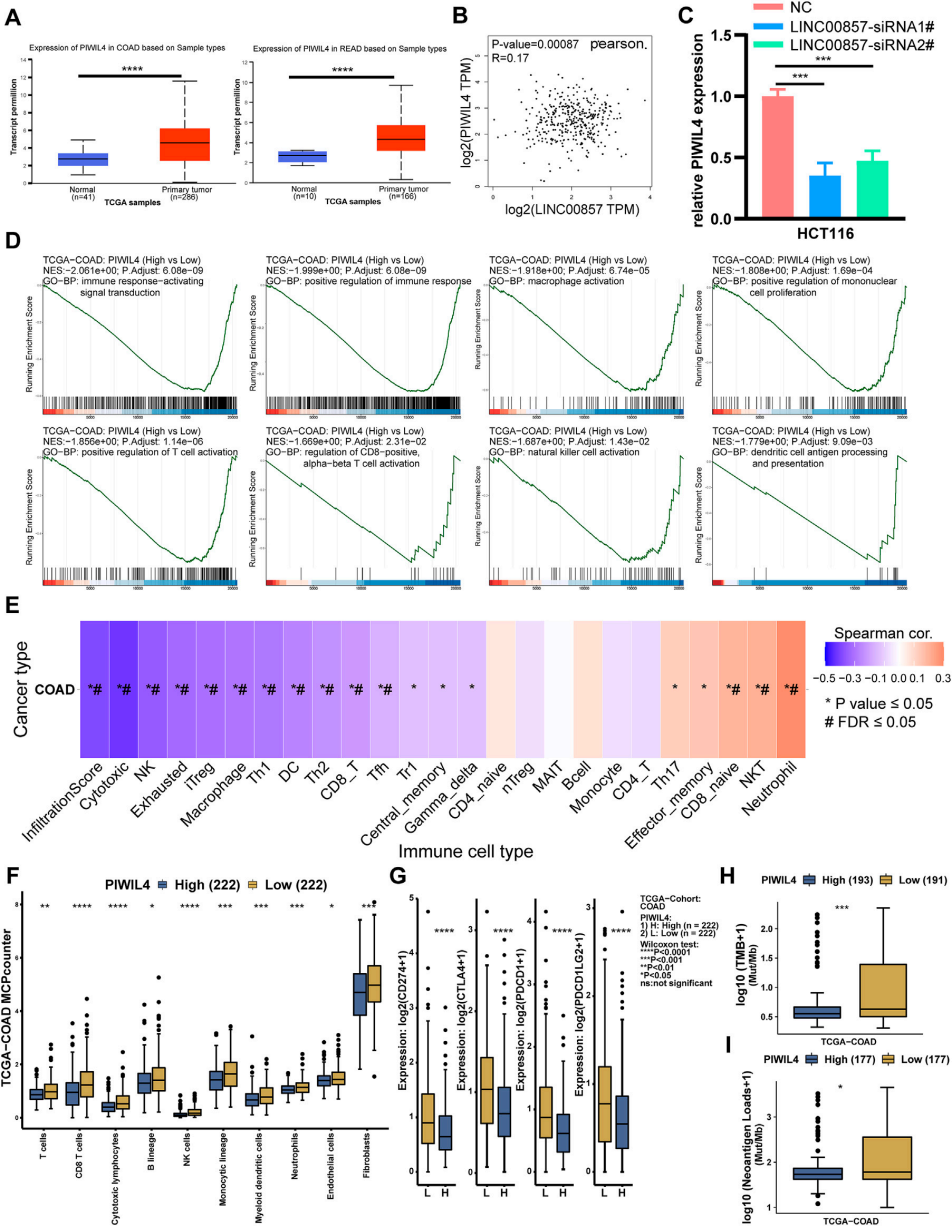
LINC00857 regulated the expression of PIWIL4 in colorectal cancer. **(A)** PIWIL4 expression in colorectal cancer samples and normal samples was explored *via* UALCAN database. **(B)** The relevance between LINC00857 and PIWIL4 expression in colorectal cancer was explored *via* GEPIA2 database. **(C)** The expression of PIWIL4 mRNA after LINC00857 silencing was determined *via* qPCR assay. **(D)** GSEA of the relevance between PIWIL4 expression and immune response pathways in COAD. (|NES|>1, *p* adjust <0.05) **(E)** The correlation between GSVA score and immune infiltration in colon cancer. (*p value < 0.05, #FDR<0.05). **(F–I)** Differences of immune cell infiltration levels, immune checkpoint genes expression levels, TMB and NEO status between PIWIL4 high and low expression group.

To pinpoint the downstream target genes of LINC00857, we integrated two siRNA sequencing data and screened out the top five genes with the largest fold changes among all downregulated genes: RPL17-C18orf32, TREX1, TBC1D28, TEN1 and PIWIL4 (Supplementary Figure S5A). Among them, only PIWIL4 was markedly overexpressed in colorectal cancer and positively related with LINC00857 expression by bioinformatic analysis (Figures 11A,B). In Figure 11C, qPCR analysis demonstrated the expression of PIWIL4 mRNA was dramatically decreased after LINC00857 knockdown. These data validated that PIWIL4 was a target of LINC00857 in colorectal cancer. To probe the possible roles of PIWIL4 in colorectal cancer, we implemented GSEA of TCGA datasets according to the PIWIL4 expression status in COAD tissues. As shown in Figure 11D, GSEA results revealed that immune response-related genes were predominantly enriched in the PIWIL4 hypo-expression group. These specific pathways contain immune response-activated signal transduction, positive regulation of immune response/mononuclear cell proliferation/T cell activation, macrophage/natural killer cell activation, regulation of CD8−positive, alpha−beta T cell activation and dendritic cell antigen processing and presentation. GSVA also indicated that PIWIL4 expression was negatively related to infiltration score and immune cells such as NK, DC, CD8+T cells and macrophages in COAD (Figure 11E). PIWIL4 expression in COAD was negatively correlated with immune cell infiltration and immune checkpoint gene expression such as PD1, PD-L1, CTLA4 and PD-L2 (PDCD1LG2, programmed cell death 1 ligand 2) (Figures 11F,G; Supplementary Figures S5B,C). As anticipated, PIWIL4 expression was also inversely correlated with TMB, MSI and neoantigen in COAD (Figures 11H,I; Supplementary Figure S5D). The foregoing analysis elucidated that LINC00857 might affect the immune characteristics of colorectal cancer by regulating PIWIL4 expression.

### PIWIL4 predicted immunotherapy efficacy

**FIGURE 12:**
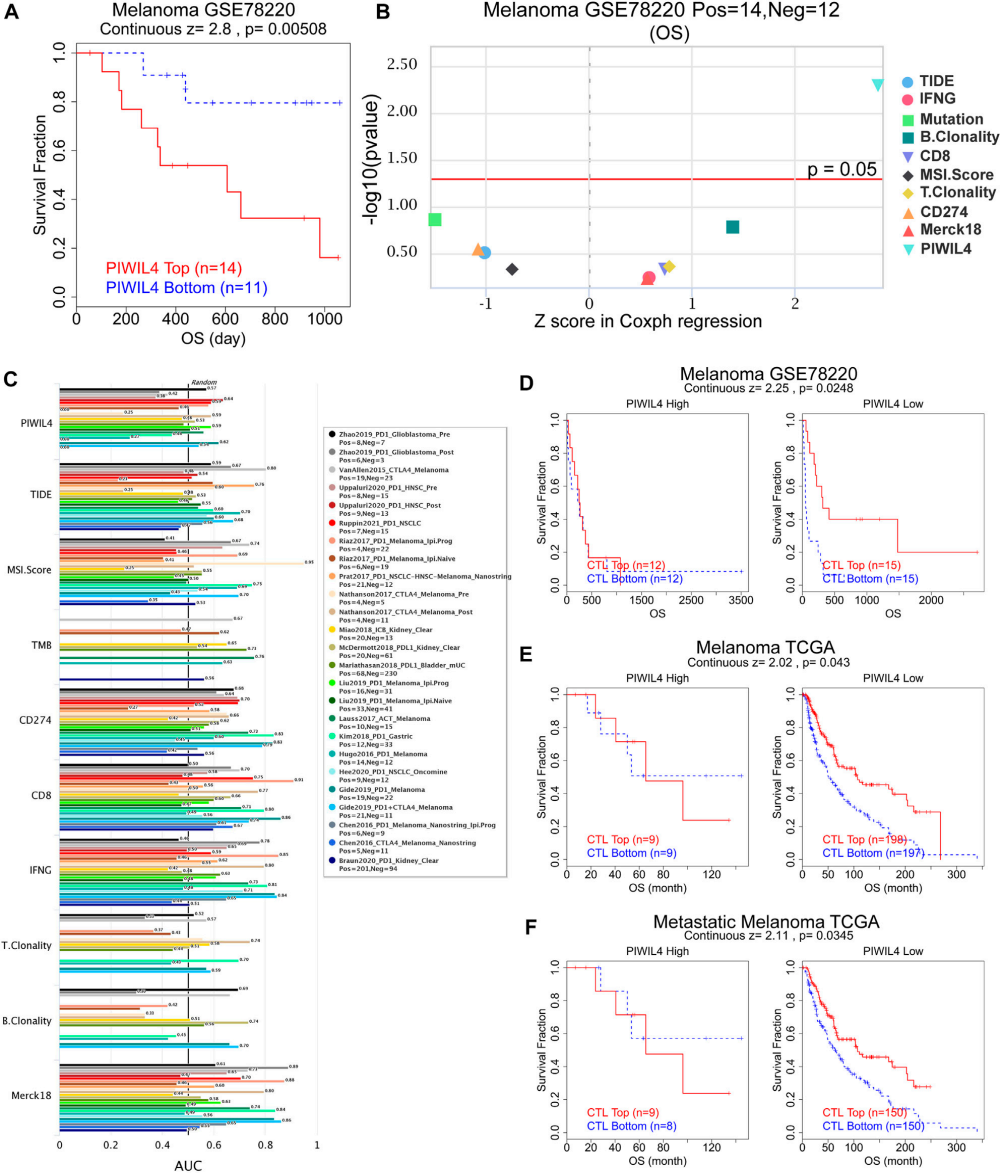
PIWIL4 predicted immunotherapy efficacy. **(A)** The overall survival analysis of patients in melanoma when receiving PD1 inhibitor. **(B)** Z score in Coxph regression analysis of different predictors of immunotherapy in melanoma. **(C)** The predictive capability of PIWIL4 for immunotherapy efficacy in multiple human immunotherapy cohorts. **(D–F)** The overall survival analysis of patients in melanoma with different PIWIL4 expression and CTL levels.

Subsequently, we assessed whether PIWIL4 expression could predict the effect of cancer immunotherapy by the TIDE platform (Fu et al., 2020). Figure 12A suggested that the patients with high-expressed PIWIL4 received poorer PD1 inhibitor treatment response. We estimated the predictive value of PIWIL4 as a biomarker for cancer immunotherapy response. As illustrated in Figure 12B, PIWIL4 could independently serve as a predictor of immunotherapy response. Compared with existing biomarkers, PIWIL4 provided higher predictive power than B. clonality, T. clonality and TMB (Figure 12C) (AUC values >0.5 means that the algorithm outperformed random). Additionally, we found hypo-expressed PIWIL4 facilitated CTL (Cytotoxic T cell)-mediated anti-tumor response, implying its potential as a novel target of immunotherapy (Figures 12D–F). In summary, these data indicated that PIWIL4 could serve as a predictor for immunotherapy response and a new target for immunotherapy.

### Susceptible drugs estimation for PIWIL4 protein

**FIGURE 13:**
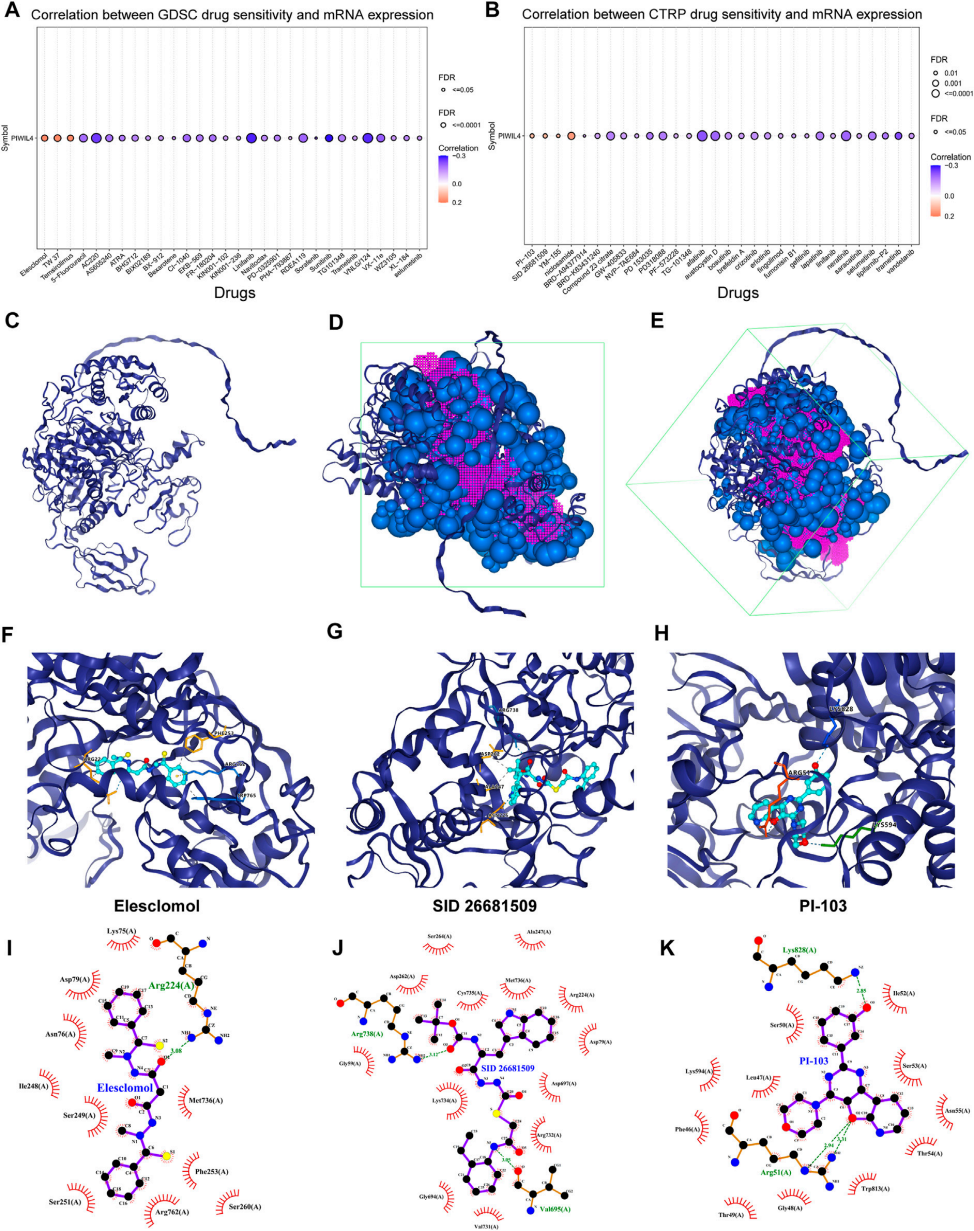
Susceptible drugs estimation for PIWIL4 protein. **(A,B)** Predicting the relevance between PIWIL4 expression and different drug sensitivities based on GDSC and CTRP databases. **(C)** The spatial structure diagram of the PIWIL4 protein. **(D,E)** Potential binding sites and box on PIWIL4 protein. **(F–H)** The optimal docking space conformation of the drug to PIWIL4 protein. (Blue dotted line: Hydrogen bond; grey dotted line: Hydrophobic interaction; yellow dotted line: Cation interaction). **(I–K)** The interaction force between PIWIL4 protein and drugs was calculated by ligplus software.

Lastly, based on the GDSC and CTRP databases, we predicted the drugs that possibly affect the function of PIWIL4 protein. The GDSC data set demonstrated that the sensitivity of Elesclomol, TW 37 and Temsirolimus was positively correlated with PIWIL4 expression (Figure 13A). Analogously, the results derived from the CTRP dataset elucidated that the sensitivity of PI-103, SID 26681509, YM-155 and niclosamide was positively correlated with PIWIL4 expression (Figure 13B). Significantly, the relevance between PIWIL4 expression and these drug activities could be verified by Cell Miner CDB online platform (Luna et al., 2021) (Supplementary Figure S6). In addition, molecular docking was performed to verify the binding abilities of these drugs with PIWIL4 protein. Concretely, the PIWIL4 protein spatial structure was displayed in Figure 13C. Putative binding sites and box on PIWIL4 protein were indicated in Figures 13D,E. Figures 13F–K and Supplementary Figure S7 illustrated the optimal docking space conformation and interaction force of each drug to PIWIL4 protein. The detailed docking scoring results were provided in Supplementary Table S1. Obviously, SID 26681509 had the best binding conformations to PIWIL4 protein. To further assess the influences of PIWIL4 expression on drug sensitivity, we examined the relationship between multiple drug activities and PIWIL4 expression in NCI-60 cancer cell lines (Supplementary Figures S8, S9). Apparently, as PIWIL4 expression elevated, the IC50 of INK-128, entosplenitib and Everolimus against cancer cells was lower. Therefore, we screened out several susceptible drugs targeting PIWIL4 protein.

### LINC00857-miRNA-PIWIL4 ceRNA network

LncRNAs in the nucleus are mainly engaged in transcriptional regulation whereas cytoplasm lncRNAs could govern the stability and translation of mRNA *via* ceRNA mechanism (Yao et al., 2019). Since FISH assays presented that LINC00857 localized mainly in the cytoplasm, we predicted many miRNAs responsible for LINC00857-mediated PIWIL4 expression by means of online database analysis. We constructed the LINC00857-miRNA-PIWIL4 network using Cytoscape software (Supplementary Figure S10A). Concretely, we used the miRNA Walk tool (Sticht et al., 2018) with the 3′UTR (untranslated region) of PIWIL4 mRNA as the query sequence to identify miRNAs that may regulate the target genes. A total of 132 LINC00857-miRNA regulatory pairs were evaluated *via* the lncRNASNP2 platform (Miao et al., 2018). Eventually, we confirmed 7 potential microRNAs including hsa-miR-6728-3p, hsa-miR-22-5p, hsa-miR-342-5p, hsa-miR-512-5p, hsa-miR-939-3p, hsa-miR-6742-3p, hsa-miR-4433b-3p. Further analysis indicated that only hsa-miR-22-5p, hsa-miR-342-5p and hsa-miR-6728-3p had potential binding sites to both LINC00857 and PIWIL4 3′UTR (untranslated region) (Supplementary Figure S10B).

## Discussion

Multiple studies have reported that LINC00857 has a high relevance with the development and progression of diverse cancers. Silencing LINC00857 alleviated the proliferative capacity and facilitated the apoptosis, autophagy and radiotherapy sensitivity of lung cancer cells, suggesting that LINC00857 could act as a potential therapeutic target for LUAD (Han et al., 2020; Su et al., 2020). Likewise, LINC00857 accelerated ovarian cancer progression and glycolysis by regulating the Hippo signaling pathway (Lin et al., 2020). So far, all studies have been limited to the effect of LINC00857 on a single cancer, but holistic analysis in multiple cancers is lacking. Pan-cancer analysis could provide insights and potential treatment strategies for cancers by comparing the similarities and differences between diverse cancers. This paper provided the first comprehensive analysis of LINC00857 expression, prognostic value as well as immune characteristics by pan-cancer datasets.

We demonstrated that LINC00857 could potentially act as a diagnostic biomarker in eight cancers, including COAD, CHOL, KIRP, HNSC, LUAD, LIHC, PAAD and STAD. Among them, LINC00857 may also serve as a prognostic biomarker in HNSC, LUAD, LIHC and PAAD. Currently, a huge challenge for cancer therapy is drug resistance, which can be greatly facilitated by the tumor microenvironment (Chatterjee and Bivona, 2019). TIICs are an integral part of the tumor microenvironment and have been shown to have a remarkable influence on the prognosis of multiple cancers (Ye et al., 2019). However, there is few research about the correlation between LINC00857 expression and tumor immunity (Mu et al., 2021). Our results suggest that LINC00857 expression was negatively associated with immune infiltrating cells in most of cancers, especially in eight LINC00857 high-expressing cancers. Similarly, our study revealed a negative correlation between LINC00857 expression and immune score in these cancers. Cancers may evade immune surveillance by utilizing immune checkpoint genes including PD-1, CTLA-4 and PD-L1 (Postow et al., 2015). Blocking these immune checkpoint genes has proven to be a valid strategy for cancer therapy (Ramagopal et al., 2017). Interestingly, our research also found that LINC00857 expression was negatively related to the expression of immune checkpoint genes including PD1, CTLA4 and PD-L1 in LINC00857 high-expressing cancers. All the data suggest that high-expression of LINC00857 was closely correlated with an immunosuppressive microenvironment.

MSI is a hypermutator phenotype due to the absence of DNA mismatch repair (Baretti and Le, 2018). Cancer patients with high microsatellite instability (MSI-H) are more likely to have a durable response to immunotherapy (Janjigian et al., 2018). MSI was found in numerous cancer types, with a high incidence in colorectal, endometrial and gastric cancer (Hause et al., 2016). Additionally, mutations in cancer cells can produce new epitopes of autoantigens, which can trigger tumor-specific T-cell response to facilitate immunotherapy (Cohen et al., 2015; Blass and Ott, 2021). At present, MSI, neoantigens (NEO) and tumor mutation burden (TMB) have become essential biomarkers for immunotherapy (Schumacher and Schreiber, 2015; Dudley et al., 2016; Yarchoan et al., 2017). Significantly, our data confirmed a negative association between LINC00857 expression and TMB/MSI/NEO in COAD, suggesting that immune checkpoint inhibitors may have poor response in those cancer patients with high-expression of LINC00857. Though LINC00857 expression was positively associated with MSI/TMB in some cancers, it may be still unsuitable for immunotherapy in consideration of the immune infiltrating cells status and immune checkpoint genes expression. In other words, the patients with high-expression of LINC00857 were not suitable for immune checkpoint inhibitors. LINC00857 may serve as a predictor for immunotherapy efficacy.

We wondered whether targeting LINC00857 could be feasible? Unsurprisingly, our data verified that LINC00857 was essential for the proliferation of colorectal cancer cells. This observation matched previous studies that LINC00857 could significantly promote the survival of cancer cells (Tang et al., 2021; Zhou et al., 2021). To further investigate the functions of LINC00857 in colorectal cancer, we conducted RNA sequencing (RNA-seq) after LINC00857 knockdown in HCT116 cells. The GSEA results hinted that LINC00857 may accelerate cancer progression by inhibiting the ferroptosis pathway in colorectal cancer. Besides, LINC00857 may also affect cancer growth by mediating lipid metabolism, glycolysis and multiple signaling pathways. The subsequent WGCNA unveiled the latent molecular mechanisms of LINC00857 in colorectal cancer. Interestingly, combining the bioinformatics and qPCR verification, we eventually discovered that LINC00857 probably exert its oncogenic effects by mediating PIWIL4 expression, which presented oncogenic potential in multiple cancer types (Su et al., 2012; Wang et al., 2016). Previous studies reported that PIWIL4 was probably a pivotal biomarker for predicting prognosis and immune landscape of cholangiocarcinoma (Zou et al., 2021). Our study further elucidated the relevance of PIWIL4 expression with the immune features of colorectal cancer, which was in accordance with the roles of LINC00857. High expression of PIWIL4 lead to poor response to immunotherapy, suggesting that PIWIL4 could also serve as a predictor of immunotherapy efficacy. Detecting the expression of LINC00857/PIWIL4 axis may contribute to screening out the exact patients suitable for immunotherapy. PIWIL4 had higher predictive power and may influence the function of CTL (Cytotoxic T cell), hinting a potential to be a new target for immunotherapy. These discoveries could likely help to illuminate the roles of LINC00857/PIWIL4 axis in colorectal tumorigenesis and development, provide a novel perspective for colorectal cancer immunotherapy, and make it possible to achieve more precise and efficient immunotherapy in the clinic. Finally, we screened out several susceptible drugs targeting PIWIL4 protein by sensitivity analysis and molecular docking analysis. This offered a powerful rationale for the clinical discovery of drugs targeting PIWIL4 for colorectal cancer treatment. Collectively, our research integrally analyzed the oncogenic roles of LINC00857 in pan-cancer. Although we have synthesized several databases for a comprehensive and systematic analysis of LINC00857, there are still some limitations and deficiencies in this research. For example, we need additional clinical data to validate our findings.

## Conclusion

LINC00857 might act as a novel diagnostic and prognostic biomarker for diverse cancers. LINC00857 expression was closely correlated with an immunosuppressive microenvironment. LINC00857/PIWIL4 axis may be predictive biomarkers for immunotherapy and valuable molecular targets for malignant tumors.

## Data Availability

The datasets presented in this study can be found in online repositories. The names of the repository/repositories and accession number(s) can be found below: Gene Expression Omnibus, GSE201069.

## Glossary

ACC: adrenocortical carcinoma
BP: biological process
BLCA: bladder urothelial carcinoma
BRCA: breast invasive carcinoma
CC: cellular component
CDCs: conventional dendritic cells
CESC: cervical squamous cell carcinoma and endocervical adenocarcinoma
CHOL: cholangiocarcinoma
COAD: colon adenocarcinoma
CTL: cytotoxic T cell
DEGs: differentially expressed genes
DFI: disease-free interval
DFS: disease free survival
DLBC: lymphoid neoplasm diffuse large B-cell lymphoma
DSS: disease specific survival
FISH: fluorescence *in situ* hybridization
GBM: glioblastoma multiforme
GO: gene ontology
GSEA: gene set enrichment analysis
HNSC: head and neck squamous cell carcinoma
KEGG: kyoto encyclopedia of genes and genomes
KICH: kidney chromophobe
KIRC: kidney renal clear cell carcinoma
KIRP: kidney renal papillary cell carcinoma
KM: Kaplan-Meier
LIHC: hepatocellular carcinoma
LUAD: lung adenocarcinoma
LUSC: lung squamous cell carcinoma
MESO: mesothelioma
MF: molecular function
MSI: microsatellite instability
ncRNAs: non-coding RNAs
NEO: neoantigen
OD: optical density
OS: overall survival
PAAD: pancreatic adenocarcinoma
PCPG: pheochromocytoma and paraganglioma
PDCD1: programmed cell death 1
PDCD1LG2: programmed cell death 1 ligand 2
PD-L1: programmed cell death 1 ligand 1
PFI: progression-free interval
PRAD: prostate adenocarcinoma
RFS: recurrence-free survival
SKCM: skin cutaneous melanoma
STAD: stomach adenocarcinoma
TCGA: the cancer genome atlas
TGCT: testicular germ cell tumors
THCA: thyroid carcinoma
TIICs: tumor-infiltrating immune cells
TMB: tumor mutation burden
UCEC: uterine corpus endometrial carcinoma
UCS: uterine carcinosarcoma
UVM: uveal melanoma
WGCNA: weighted gene co-expression network analysis
