# Supplementary material for: Exploring the oncogenic roles of LINC00857 in pan-cancer
Source: Front Pharmacol. 2022 Sep 8;13:996686. doi: 10.3389/fphar.2022.996686 (PMC9498830; doi:10.3389/fphar.2022.996686)
Supplement: Supplementary file 1 [file Image1.pdf]

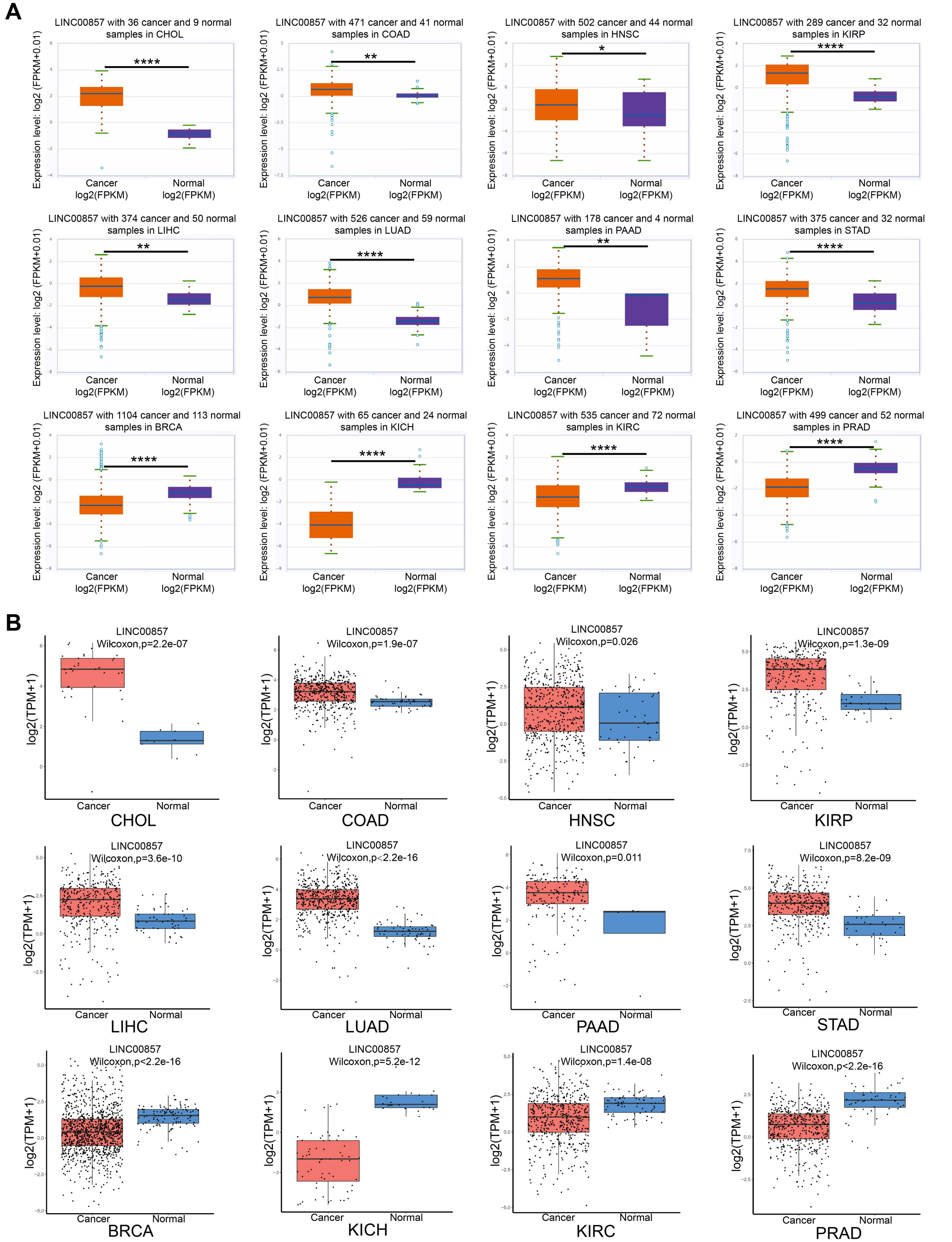

Supplementary Figure 1. The expression levels of LINC00857 in pan-cancer. **(A-B)** The expression levels of LINC00857 in different carcinomas (COAD, CHOL, HNSC, KIRP, LUAD, LIHC, PAAD, STAD, BRCA, KICH, KIRC, PRAD) and adjacent normal tissues was explored through StarBase V3.0 and Lnc2Cancer 3.0 database. (*P < 0.05; **P < 0.01; ***P < 0.001).

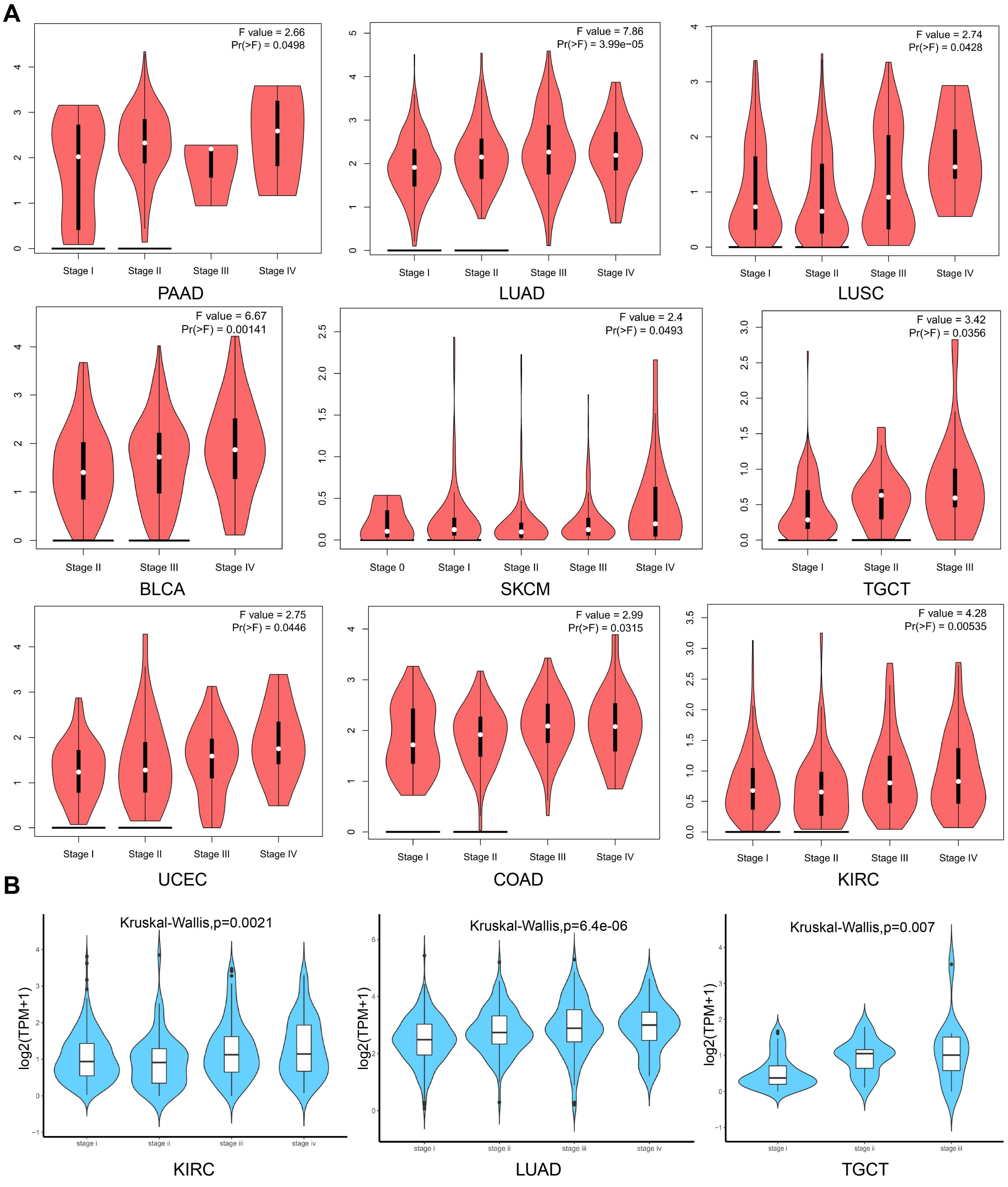

Supplementary Figure 2. Pan-cancer analysis of the relevance between LINC00857 expression and tumor stages. **(A)** The relevance between LINC00857 expression and diverse pathological stages of tumors was analyzed based on GEPIA2 database. **(B)** The pathological stage-dependent expression levels of LINC00857 in KIRC, LUAD and TGCT were estimated and compared by Lnc2Cancer 3.0 database. (*P < 0.05; **P < 0.01; ***P < 0.001).

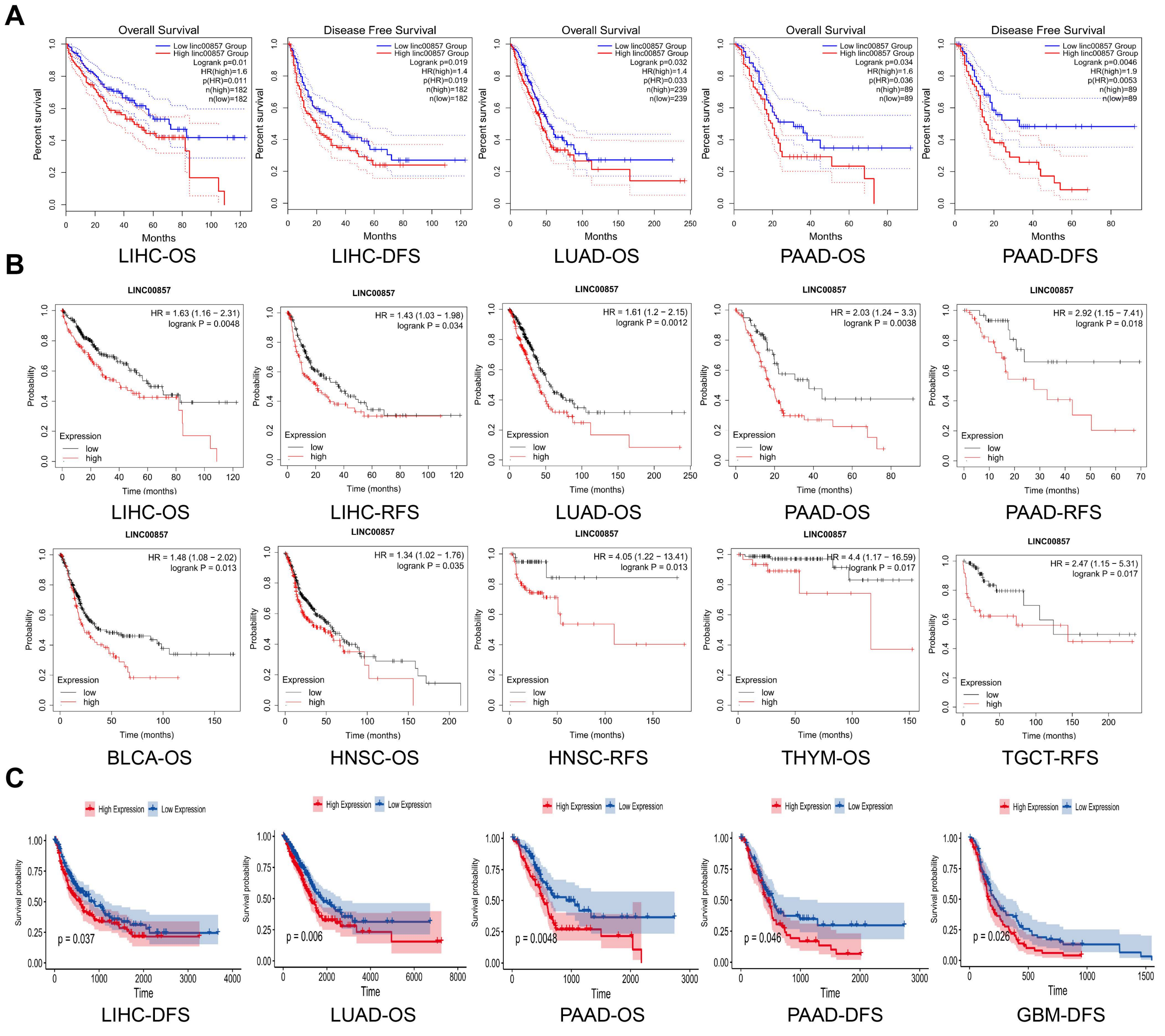

Supplementary Figure 3. The prognostic value of LINC00857 in pan-cancer. **(A)** The prognosis analysis of LINC00857 in pan-cancer using GEPIA2 database. **(B)** The prognosis analysis of LINC00857 in pan-cancer using the Kaplan-Meier Plotter. **(C)** The prognosis analysis of LINC00857 in pan-cancer using the Lnc2Cancer 3.0 database. (*P < 0.05; **P < 0.01; ***P < 0.001).

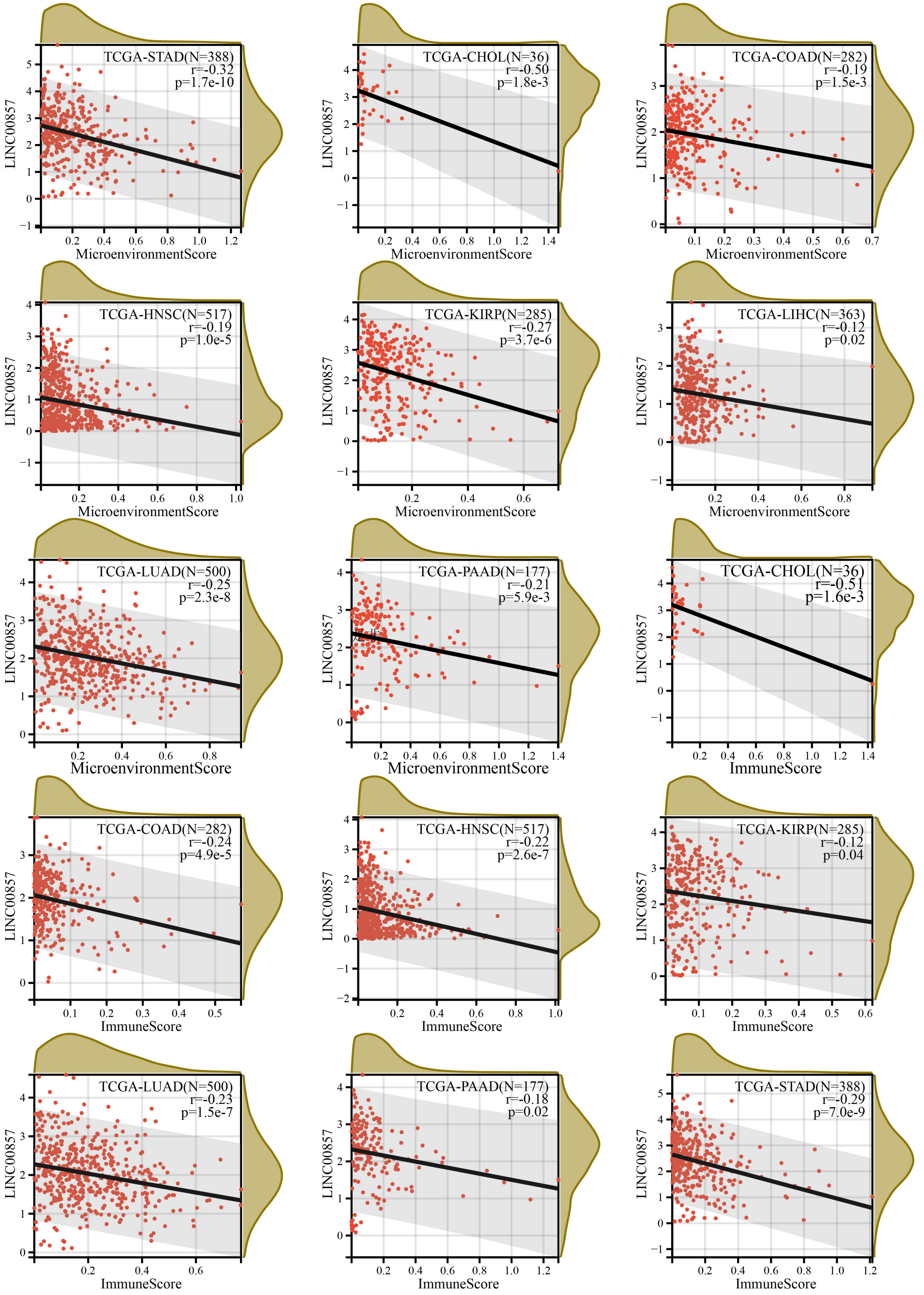

Supplementary Figure 4. The association between LINC00857 expression and immune score and microenvironment score. The scatterplot revealed the relation between LINC00857 expression and microenvironment score or immune score in HNSC, CHOL, COAD, KIRP, STAD, LIHC, PAAD and LUAD. (XCELL algorithm, Pearson analysis)

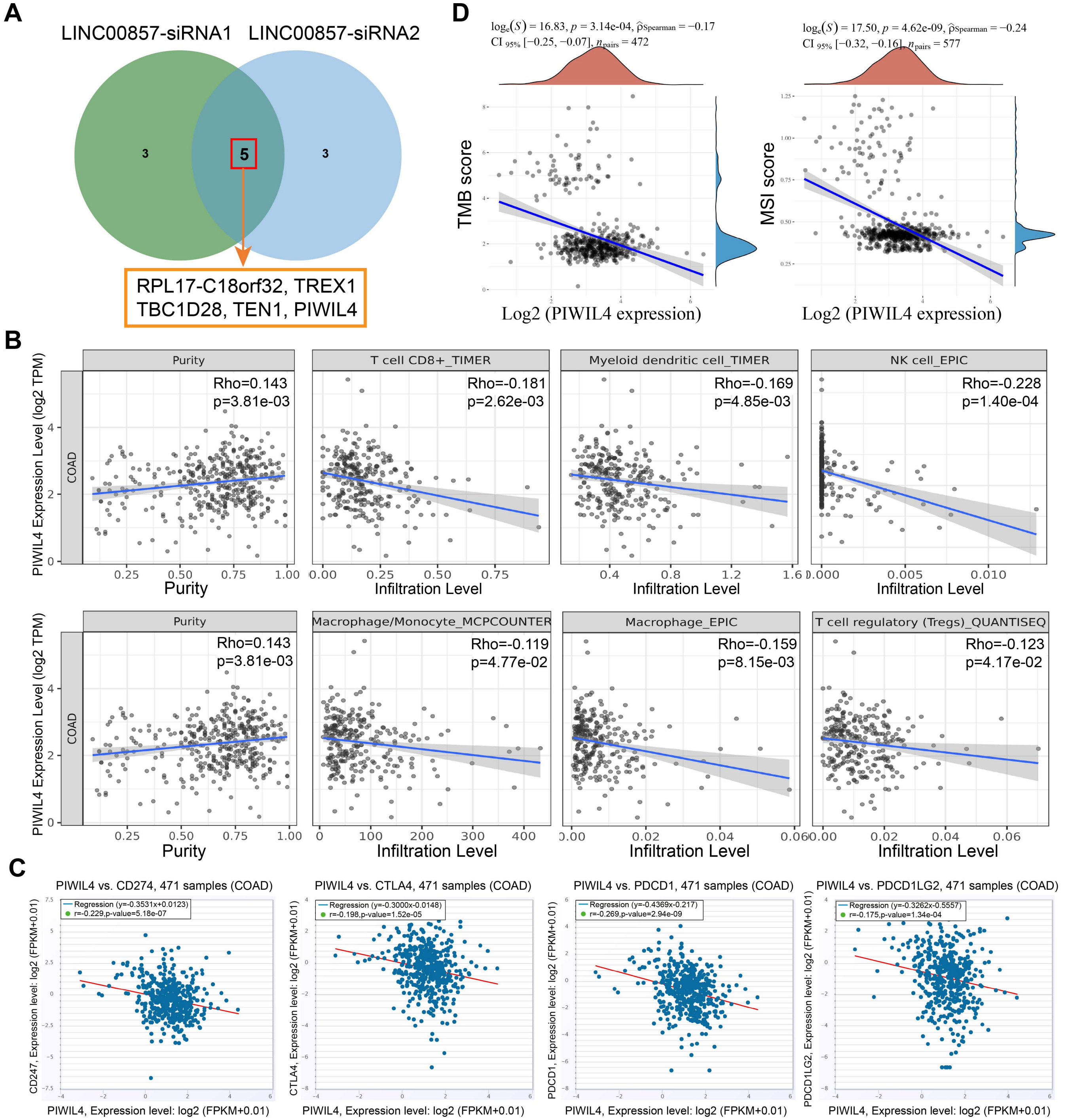

Supplementary Figure 5. Immune characteristics of PIWIL4 in colorectal cancer. **(A)** Venn diagram illustrated the intersection of the top 8 most significantly down-regulated DEGs from RNA-seq data after LINC00857 silencing. **(B)** Correlation between PIWIL4 expression and immune infiltration levels of six immune cells (myeloid dendritic cell, CD8+ T cell, macrophages/monocyte, NK cell and Treg cell) in COAD using TIMER2 database. **(C)** The correlation between PIWIL4 and PD-L1, CTLA4, PD1 and PD-L2 expression in COAD using the StarBase database. **(D)** Correlation between PIWIL4 expression and MSI or TMB in colorectal cancer.

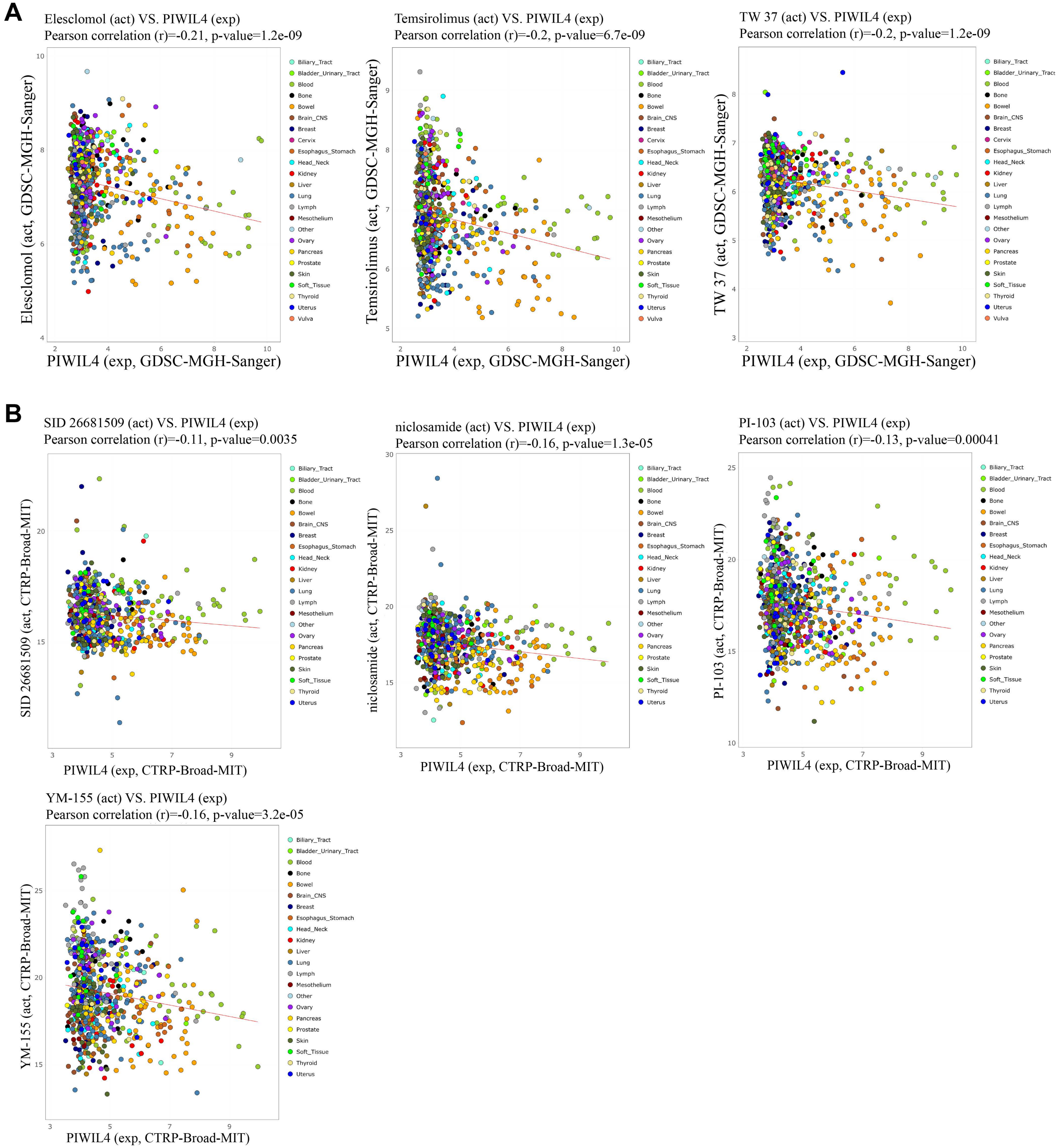

Supplementary Figure 6. The correlation between LINC00857 expression and drug activity. **(A)** The correlation between PIWIL4 expression and drug activity (-log10[IC50M]) was verified based on GDSC database. **(B)** The correlation between PIWIL4 expression and drug activity (AUC) was verified based on CTRP database. ($P < 0.05$).

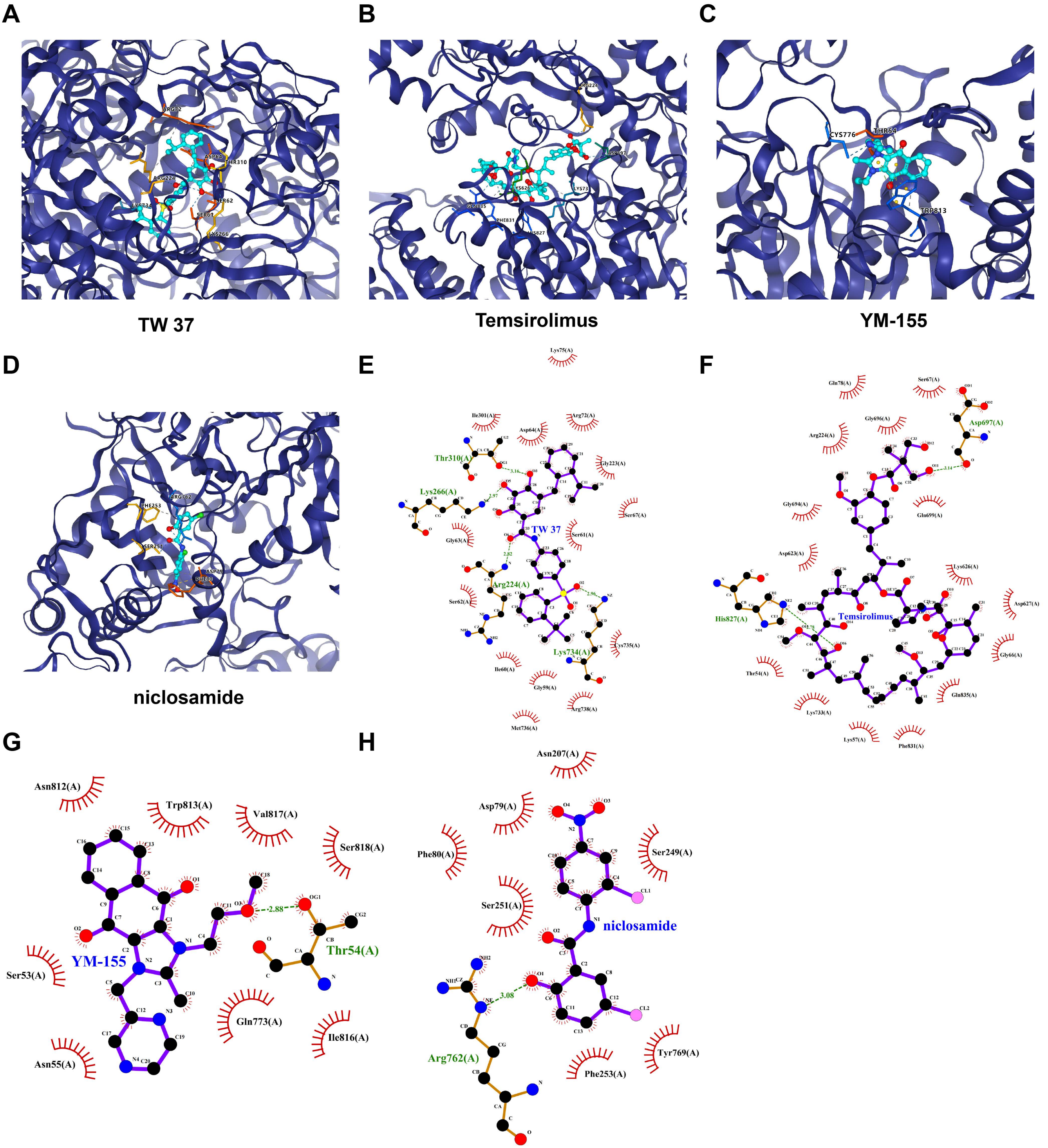

Supplementary Figure 7. Molecular docking and interaction force analysis for PIWIL4 protein. **(A-D)** The optimal docking space conformation of drugs to PIWIL4 protein. **(E-H)** The interaction force between PIWIL4 protein and drugs.

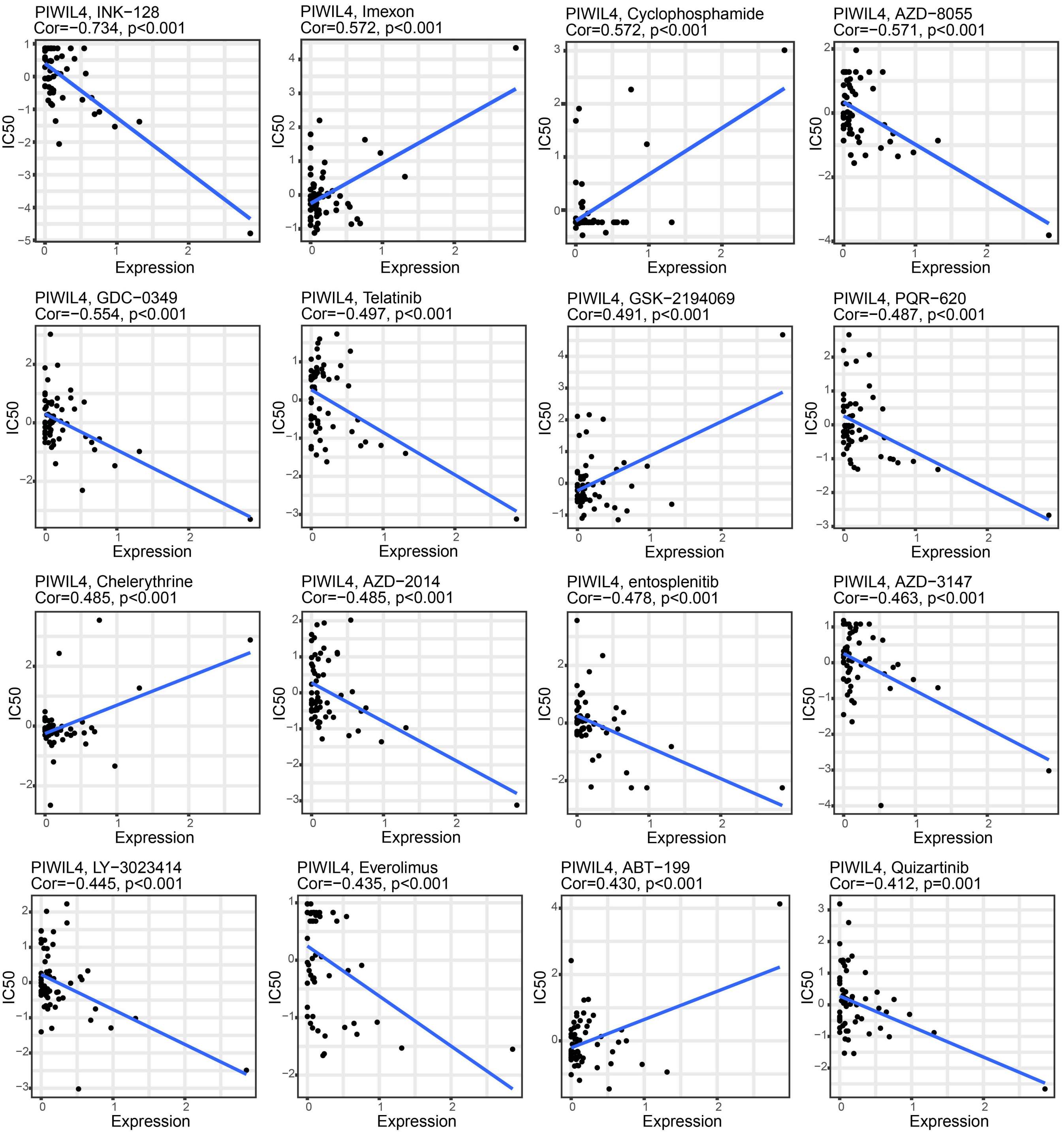

Supplementary Figure 8. The relevance between PIWIL4 expression and drug response. Only the first 16 statistically significant scatter plots were displayed. (P<0.05).

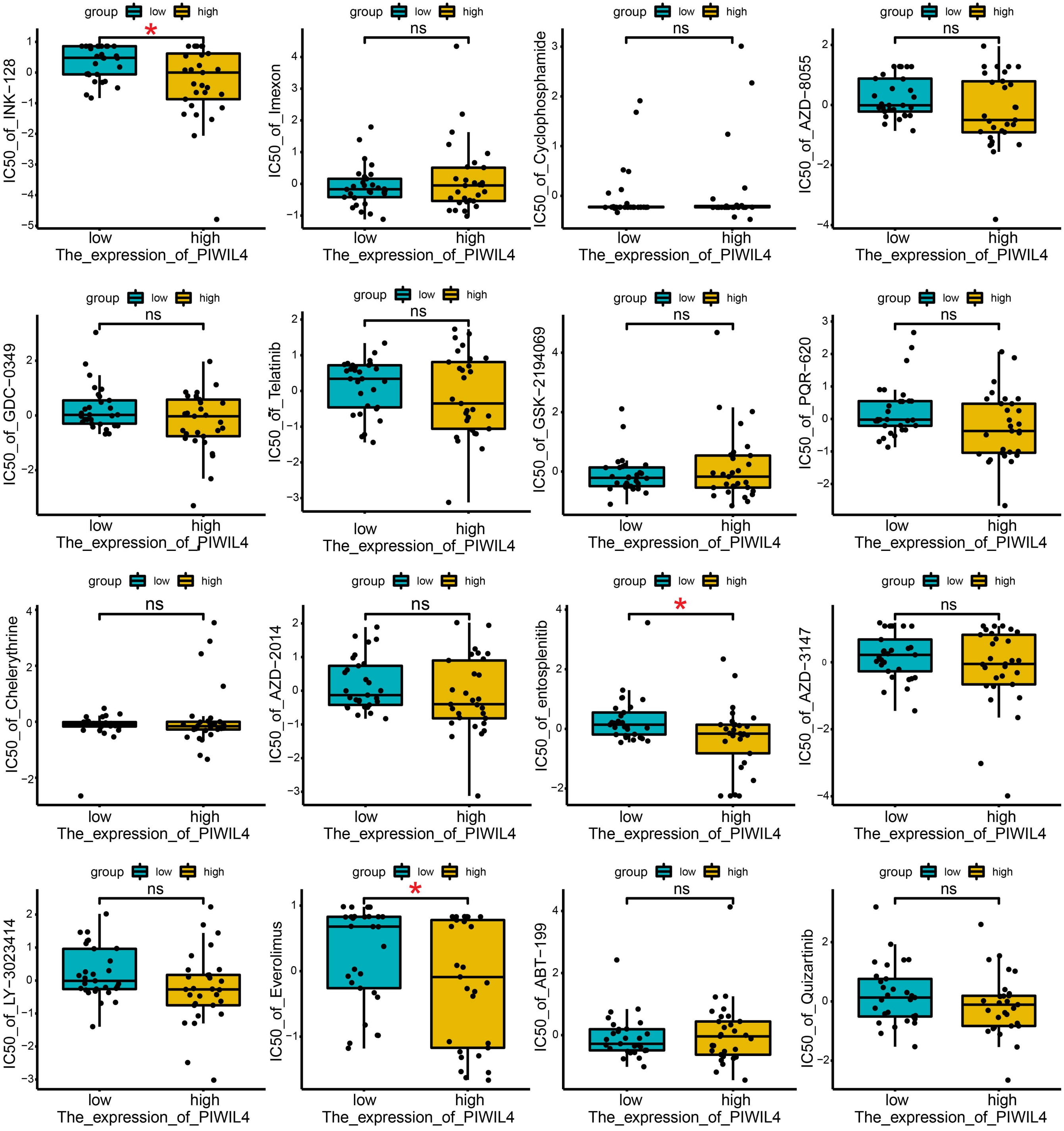

Supplementary Figure 9. Drug susceptibility in PIWIL4 high-expression and low-expression groups (*$p < 0.05$).

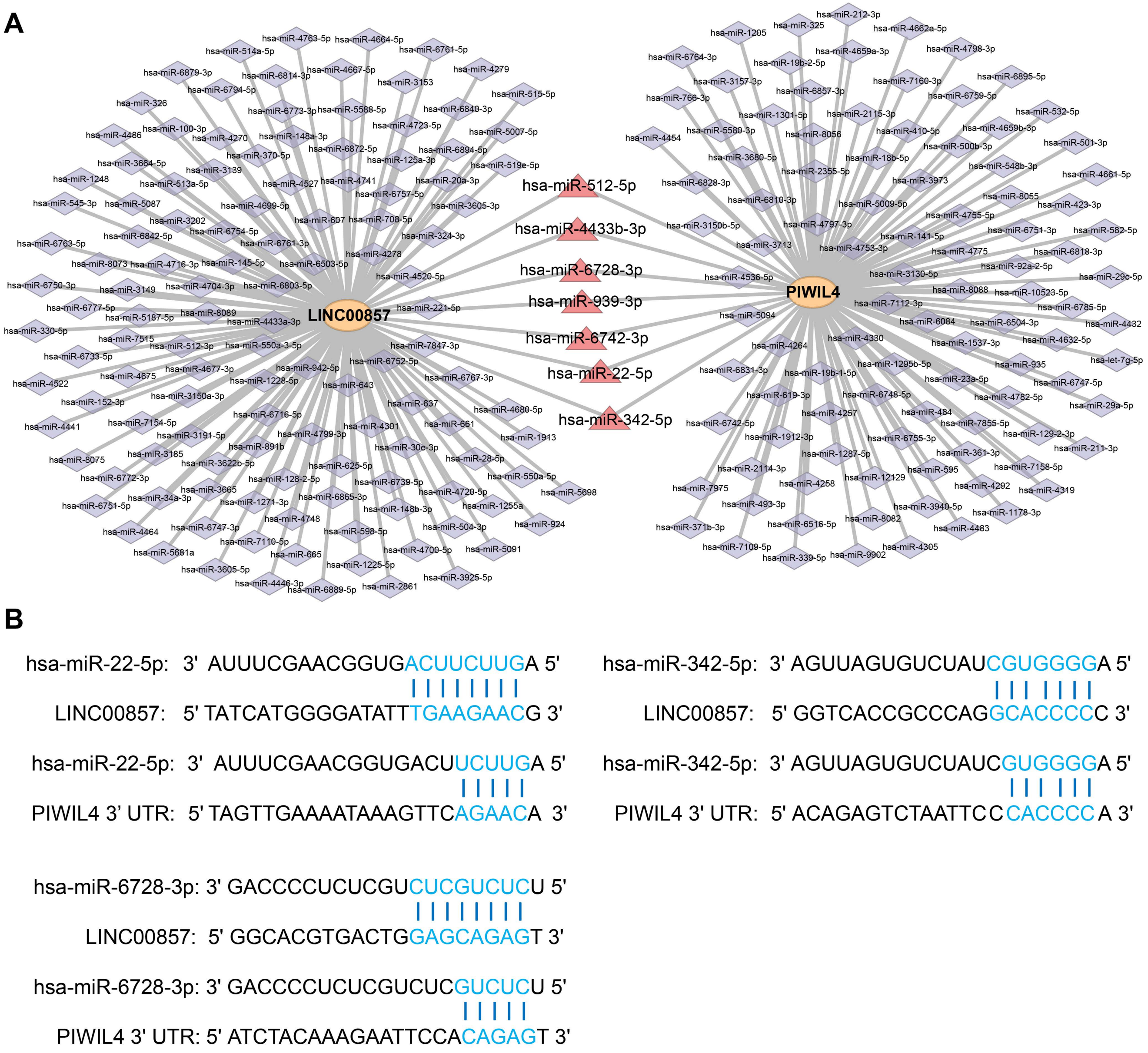

Supplementary Figure 10. LINC00857-miRNA-PIWIL4 ceRNA network. **(A)** The LINC00857-miRNA-PIWIL4 network was constructed with Cytoscape. **(B)** The putative binding sequence between PIWIL4 3’-UTR, LINC00857 and the predicted miRNA.

Table S1 PIWIL4 Docking score of seven target drugs.

Name	Pose	Grid_Score	Grid_vdw_energy	Grid_es_energy	Internal_energy_repulsive
Temsirolimus	1	-95.913567	-87.67572	-8.237844	43.538425
Temsirolimus	2	-92.408356	-88.803154	-3.605202	40.785313
Temsirolimus	3	-88.912216	-86.10257	-2.809647	37.749699
TW 37	1	-90.394104	-81.503738	-8.890368	35.861485
TW 37	2	-89.919052	-80.683342	-9.235709	81.161697
TW 37	3	-87.994873	-80.946472	-7.0484	66.817955
TW 37	4	-83.89727	-74.637756	-9.259513	72.592278
TW 37	5	-83.512894	-75.408928	-8.103968	59.224133
TW 37	6	-82.529167	-75.778603	-6.750567	63.679447
TW 37	7	-81.370789	-78.517632	-2.853155	66.448837
Elesclomol	1	-70.096832	-64.105682	-5.991151	15.393012
Elesclomol	2	-68.628632	-59.314648	-9.313986	11.916723
Elesclomol	3	-66.978401	-58.093903	-8.884501	10.673527
SID 26681509	1	-85.607452	-81.702271	-3.905184	13.009789
SID 26681509	2	-83.938019	-80.852882	-3.085136	15.068235
SID 26681509	3	-83.542625	-80.653351	-2.889277	14.406669
PI-103	1	-56.742683	-52.390713	-4.351972	4.939725
PI-103	2	-55.563366	-50.397873	-5.165492	13.225006
PI-103	3	-54.917274	-51.920425	-2.996851	4.789149
PI-103	4	-54.819466	-53.277534	-1.54193	4.033278
PI-103	5	-54.360394	-50.856113	-3.50428	4.24295
YM-155	1	-53.901466	-52.168709	-1.732757	11.413545
YM-155	2	-53.829002	-53.585552	-0.243449	12.584155
YM-155	3	-53.103405	-54.826279	1.722875	13.476639
YM-155	4	-52.535358	-55.859268	3.32391	12.41807
YM-155	5	-52.195347	-52.657501	0.462154	12.106308
YM-155	6	-51.830185	-52.942574	1.11239	11.926449
niclosamide	1	-54.670029	-48.82592	-5.84411	3.144021
